# Historical and Recent Developments in the Chemistry of Cyanate Congeners

**DOI:** 10.1021/acs.inorgchem.5c01041

**Published:** 2025-06-11

**Authors:** Stephan Hohloch, Frank Tambornino

**Affiliations:** † 27255 Leopold-Franzens-University Innsbruck, Faculty of Chemistry and Pharmacy, Institute of General, Inorganic and Theoretical Chemistry, Innrain 80-82, 6020 Innsbruck, Austria; ‡ Phillipps-Universität Marburg, Department of Chemistry, Hans-Meerwein-Strasse 4, D-35043 Marburg, Germany

## Abstract

The cyanate anion, [OCN]^−^, and its heavier congeners with the general formula [ChCPn]^−^ (Ch = O–Te, and Pn = N–As) are fundamental in introductory chemistry textbooks, chemical laboratories worldwide, and modern research. Their discovery spans more than 200 years and includes key milestones in the history of chemistry, e.g., concepts of isomerism and pseudohalogens. Today, in particular, heavy pnictogen congeners are valued as versatile building blocks for chemical transformations and the synthesis of a multitude of exciting new compounds. Given the importance of these anions in history and recent chemistry, this Review first explores the historical development of these anions starting from the very beginning of the modern chemical literature (1800s) until today. This section is followed by an in-depth summary and comparison of their electronic structures leading to their development into vital building blocks for chemical transformations. Finally, we present a comprehensive state-of-the-art overview of the chemistry of the heaviest congeners, emphasizing the vast opportunities that these fascinating anions offer to contemporary chemists.

## Graphical abstract

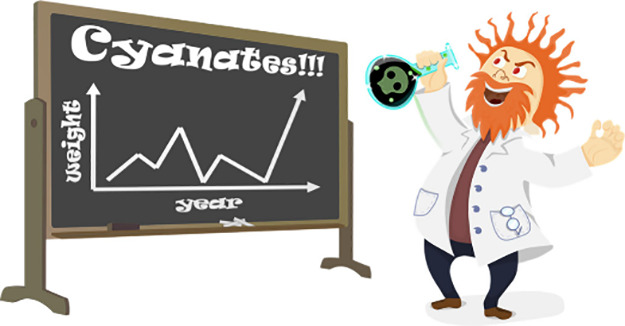

## Introduction

At its core, chemistry has always been about transformation and modification. This concept encompasses the exchange of (reactive) side groups, elongation of side chains, introduction of different functional groups, and, of course, the exchange of elements within the same group. Such modifications have led to remarkable discoveries such as the recent development of triplet nitrenes^1,2^ and bismuthidenes^3^ with similar ligands. One of the oldest stories of permutation of elements involves cyanate anions. Being archetypical pseudohalogens, cyanates have a long history of element exchange reactions. Historically, the chalcogen atoms were the first to be swapped and exchange of the oxygen atom in [OCN]^−^ for sulfur leads to thiocyanate anion [SCN]^−^. Similarly, the selenocyanate, [NCSe]^−^, and tellurocyanate, [NCTe]^−^, anions have emerged on the chemical landscape over the past two centuries. A more recent development is the extension of this exchange to the pnictogens, which started in 1992[Fn fn1] with the isolation of phosphaethynolate anion [OCP]^−^. Unlike the chalcogenide series, not all pnictogen homologues have been realized. The arsaethynolate anion [OCAs]^−^ is currently the heaviest known congener. Antimony- or bismuth-derived cyanates are still unknown. Finally, both substitution patterns can be combined, leading to the combined heavy group 15/16 homologues, i.e., phosphaethynthiolate [SCP]^−^, phosphaethynselenolate [SeCP]^−^, arsaethynthiolate [SCAs]^−^, and arsaethynselenolate [SeCAs]^−^. The discovery of these cyanate congeners spans more than two centuries and continues to develop, with new representatives still emerging.

Historically, cyanates have played pivotal roles in the development of chemical theories and understanding. Scholars often regard “Wöhler’s urea synthesis” from NH_4_OCN as the first example of synthesizing an “organic” compound from “inorganic” starting materials.^4^ At the time, any substance that could not be created without living organisms was classified as “organic”. This made urea a classical example, as it could only be made from urine. In contrast, “salts” were typical inorganic substances. When Wöhler attempted to synthesize ammonium cyanate, the reaction instead produced pure urea.^4^

This groundbreaking “inorganic-to-organic” synthesis, however, was not the first of its kind. It was preceded by the less known synthesis of oxalic acid, also performed by Wöhler, in 1824.^5,6^ However, this work failed to gain widespread attention, and “Wöhler’s urea synthesis” remains the iconic milestone in the history of chemistry. Later, Wöhler and Liebig successfully synthesized ammonium cyanate, whose crystal structure was elucidated nearly 150 years later.^7,8^

Another pivotal development was the formulation of the concept of “isomerism”, closely tied to the cyanate ion. Wöhler and Liebig discovered that silver cyanate^9^ and silver fulminate^10–12^ shared the same elemental composition but behaved differently. Silver cyanate merely burns when ignited, but silver fulminate detonates. At the time, both researchers initially suspected errors in each other’s analyses. However, they ultimately agreed with each other’s findings. Berzelius reviewed their research and coined the term “isomerism” as a chemical principle.^13^

Intimately connected to cyanates is the pseudohalogen concept. The term was coined by Birckenbach and Kellermann in 1925, to avoid the use of the term “radical”, which could have a different meaning at the time.^14^ As [CN]^•^ behaves similarly to a halogen atom, they specified the term “pseudohalogen”. In a broader sense, all pseudohalogens share the following properties. (1) The free monoradical has a strong electron affinity. (2) The free anion is a stable species. (3) Protonation gives the respective acid. (4) Low-solubility salts are formed with silver, lead, and mercury. (5) Oxidation of the anions leads to the neutral dipseudohalogen, which disproportionates in aqueous alkaline media. (6) Similarly, interpseudohalogen species as well as halogen–pseudohalogen species are known. (7) More recently, it was observed that pseudohalonium ions can be formed.^15^

This Review is divided into three parts. The first part outlines the historical development of the seminal syntheses of various cyanate anions, structured into different epochs based on historical time lines. Epoch 1 covers the early discoveries of [OCN]^−^, [SCN]^−^, and [SeCN]^−^. For Epoch 2, structure elucidation and the discovery of [TeCN]^−^ are discussed. Epoch 3 spans the past 30 years, highlighting the synthesis of the respective heavy pnictogen homologues. Next, the electronic structures of the cyanate congeners are discussed, exploring their impact on bonding and nuclear magnetic resonance properties. The third part presents a comprehensive state-of-the-art discussion on the chemistry of the heavy pnictogen congeners, excluding [OCP]^−^, which has been reviewed recently.^16^

## Results and Discussion

### Part I: Historical Developments

Generally, there are three epochs that can be distinguished. (I) The nitrogen-containing congeners, which are the cyanate itself alongside thiocyanate and selenocyanate, were first discovered. (II) After the discovery of X-ray diffraction, the simple salts were characterized by crystallographic methods, clearing up, or proving, their constitution. Concurrently, the discovery of the tellurocyanate anion unfolds, culminating in its crystallographic characterization. (III) Third was the seminal synthesis of the first heavy pnictogen homologue, the phosphaethynolate anion, and other congeners. Today, simple and stable [OCN]^−^ and [SCN]^−^ are ubiquitous in chemistry, whereas [OCP]^−^ has developed into an established component in molecular inorganic chemistry. The other heavy homologues, whether it be [SeCN]^−^, [TeCN]^−^, or the other group 15 congeners, are still niche anions with few synthetic applications.

#### Epoch 1: Early Discoveries

Due to the lack of a mature atomistic theory and chemical bonding, 18th century chemical texts are difficult for modern chemists to comprehend. Many concepts that we now take for granted had not yet been developed at that time. Dalton’s atomistic theory was gaining traction; however, it was also disputed.^17^ Substances were thought to react to different substances, although their inner workings of chemical bonding were unknown. Some terminology has shifted its meaning since then. For example, cyanogen is often mentioned as the “radical” of hydrogen cyanide. Adding further complexity is the use of different languages, such as English, German, French, and Swedish, each of which partly promotes its own school of thinking. Within this section, we will try to put reactions and deduced findings into modern formalism and wherever necessary refer to the original wording.

Hydrogen cyanide was first described in 1752 by Pierre-Joseph Macquer (1718–1784).^18,19^ It was synthesized by a series of reactions starting from Prussian blue Fe_4_[Fe­(CN)_6_]_3_ (also called Berlin blue, Parisian blue, etc.) and subsequently received the name “prussic acid”.[Fn fn2] These reactions were repeated by Carl Wilhelm Scheele (1742–1786), who in 1782 reported that the previous findings were correct.^19^

The time line for the seminal syntheses of [OCN]^−^, [SCN]^−^, and [NCSe]^−^ is shown in Figure 1. The first cyanate congener to be synthesized was the thiocyanate anion. Its seminal synthesis is attributed to Robert Porrett (1783–1868) in 1809 (see Figure 2).^20,21^ As was often the case, the synthesis was not a targeted approach but rather serendipitous. The exact composition of hydrogen cyanide was still disputed in the early 19th century, especially whether oxygen was one of its component parts. To address these questions, Porrett aimed to synthesize potassium ferricyanide (K_3_[Fe­(CN)_6_], also called “ferruetted chyazate”) from Prussian blue by way of introducing potash and sulfur into the reaction mixture, the latter to precipitate iron.[Fn fn3] The reaction mixture consisted of Prussian blue, potassium sulfide (“sulfuret of potash”), and water. Boiling of the mixture for some time led, after filtering, to a nearly colorless filtrate. When poured into a solution of iron sulfate, the solution turned deep red, a reaction still used in undergraduate laboratory for the detection of iron. Drying and extracting the former reaction mixture with ethanol followed by evaporation of the alcohol give a salt (presumably K­[NCS]) in a pure state. It is further explained that the salt must also have a free acid (H-NCS) that can be set free by combining K­[NCS] and sulfuric acid. He named the acid “prussous acid” and its salts “prussites”; however, in a later work, he also used “red tinging acid” for obvious reasons.[Fn fn4]

**Figure 1:**
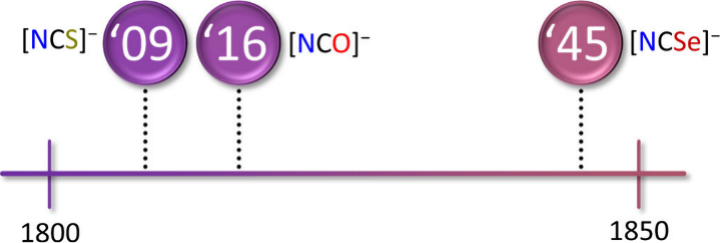
Time line of the seminal syntheses of cyanate, thiocyanate, and selenocyanate anions. For details see Epoch 1: Early Discoveries.

**Figure 2:**
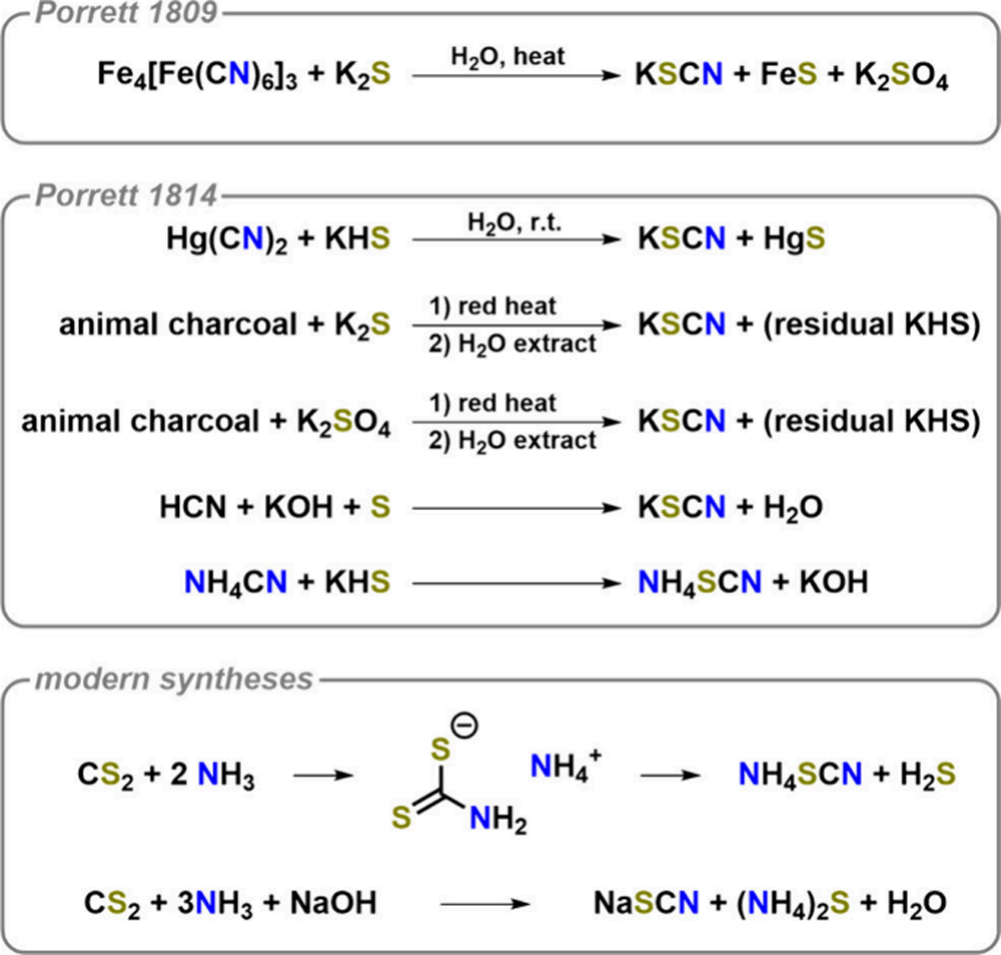
Seminal syntheses of thiocyanates by Porrett and modern industrial scale syntheses.

In another part of Europe, Joseph Louis Gay-Lussac (1778–1850) was researching the reactivity of hydrogen cyanide and cyanogen. In 1816, he had already decomposed mercury cyanide (Hg­(CN)_2_) to elementary mercury and cyanogen (CN)_2_.[Fn fn5] ^,^ ^22^

In the work first describing the cyanate ion, he focuses on elementary transformations of cyanogen. In his treatise, he writes about the reaction of cyanogen and water with minium (Pb_3_O_4_) and manganese dioxide (MnO_2_) (see Figure 3). While detailed experimental procedures are not disclosed, the general observations are cyanogen is “caught” by the oxides and cannot be smelled any more.[Fn fn6] Reactions with the resulting filtrate did not indicate the formation of a known compound. Instead, he assumes to have discovered cyanuric acid (“Blaustoffsäure”), that is H­[OCN], probably as one of its salts.^22^

**Figure 3:**
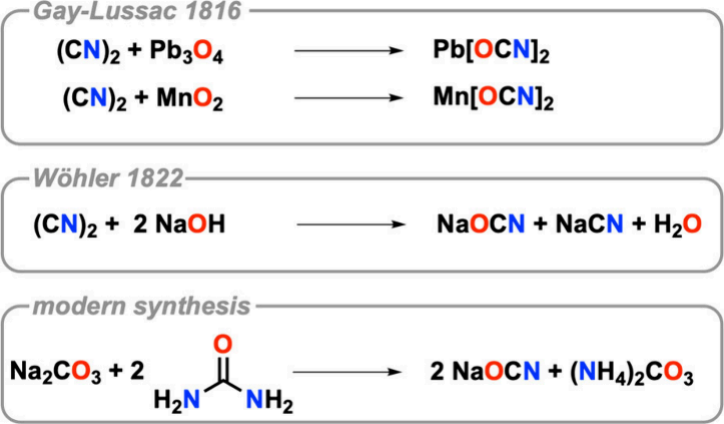
Seminal syntheses of cyanates. In the case of Pb_3_O_4_, only the reaction product of interest is shown. No side products have been characterized or indicated in the original literature.

These experiments were subsequently picked up by Friedrich Wöhler (1800–1882).^23^ He noted that toward alkaline solutions cyanogen behaves similarly to chlorine. Cyanide and cyanate salts are formed in equal parts. Our modern interpretation as “pseudohalides” sees its first spark here, although the term was coined much later.^14^ However, the separation of cyanide and cyanate was impossible. The experiments focused on barium cyanate, which was gained through reaction of cyanogen with a barium hydroxide solution. From this, he gains a series of cyanate salts (e.g., silver and alkali metals) that are described and also synthesizes an aqueous solution of H­[OCN].^23^

After the discovery of selenium in 1817,^24^ chemists soon observed that it behaves similarly to sulfur, an observation that was unexpected at the time but is now textbook knowledge. The first mention of a selenocyanate compound dates to 1845 and was published by Jöns Jakob Berzelius (1779–1848).^25^ Selenium was melted together with Prussian blue, and potassium selenocyanate can be isolated by extraction (see Figure 4). Berzelius also noted that potassium selenocyanate can be synthesized by boiling a concentrated solution of potassium cyanide with excess selenium. He briefly described its properties. K­[SeCN] crystallizes without water, can be heated to red heat without decomposition, and reacts with acids to precipitate red selenium.^25^

**Figure 4:**
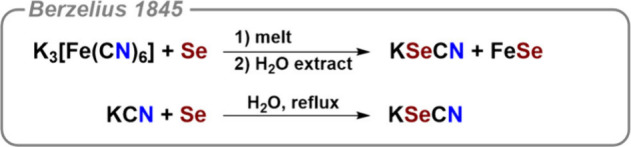
Seminal synthesis of selenocyanates by Berzelius in 1845. No side products have been characterized or indicated in the original literature.

This work was further developed by William Crookes (1832–1919) a few years later.^26^ With advancements in chemistry and a better understanding of the underlying principles, Crookes distinguished between selenocyanate salts and the free acid, avoiding older, vague terminology like “principle”. Using atomistic theory and the emerging system of element symbols, he employed an early form of modern chemical language. His elemental analyses were accurate, but the derived formulas, e.g., “KC_2_NSe_2_” instead of KNCSe, are based on a different understanding of atomic weights.[Fn fn7]

By the turn of the century, chemistry had advanced significantly across nearly all areas of the discipline. Atomistic theory was universally used; composition and structure were used to explain reactivity, and chemistry had been divided into numerous subdisciplines.

#### Epoch 2: Emergence of Chemical Concepts and X-ray Diffraction

At the beginning of the 20th century, chemistry as a whole was notably better developed. Atomistic theory was generally accepted, and subatomic particles (proton, electron, and neutron) were discovered. Whereas the early 19th century saw the emergence of the valence concept and rules regarding the proportions of elements in substances, a general understanding of chemical bonding still lacked substance. The 20th century, in contrast, thrived on the development of Lewis structures and molecular orbital theory and the concept of bonds being formed by shared electron pairs.

Concurrently, the development of X-ray crystallography allowed for the determination of the atom positions within a crystal. These studies provided concrete evidence for the existence of ionic and covalent bonds, further supporting the advances in theory of chemical bonding. The time line showing landmark syntheses and crystallographic studies is shown in Figure 5.

**Figure 5:**
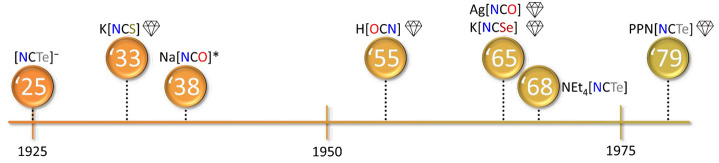
Time line of the seminal synthesis of [NCTe]^−^ and the first crystallographic studies (indicated by diamonds) of [NCO]^−^, [NCS]^−^, [NCSe]^−^, and [NCTe]^−^. *Originally, for Na­[NCO], a non-disordered structure was published. Later it was shown that the anion is head-to-tail disordered. For details see Epoch 2: Emergence of Chemical Concepts and X-ray Diffraction.

The first crystal structure to be elucidated that featured a cyanate anion was that of K­[OCN], determined in 1925.^27^ Linus Pauling (1901–1994) demonstrated that distinct K­[OCN] molecules are not present; rather, the oxygen, carbon, and nitrogen atoms are arranged in linear triatomic groups with interatomic distances of 1.16 Å. It was observed that at room temperature the cyanate anion exhibits head-to-tail disorder, preventing the differentiation between the oxygen and nitrogen termini.

The first reported non-disordered structure of a binary cyanate was published in 1938.^28^ However, it was later demonstrated that yet again the head-to-tail disordered model is the accurate representation of its structure.^29^ The earliest example of a truly non-disordered inorganic cyanate structure is likely that of Ag­[OCN] (excluding H­[OCN],^30^ where the position of the hydrogen atom was not determined with sufficient reliability).^31^

In 1933, the crystal structure of K­[SCN] was determined, marking the first example of a binary thiocyanate.^32^ Due to the similar composition to cyanates, it was initially assumed that the structures would be isotypical. Although they share similar packing motifs, K­[SCN] and K­[OCN] crystallize in different crystal systems. Like the [OCN]^−^ anion, the [SCN]^−^ anion was found to be linear. However, the quality of the data at the time was insufficient to distinguish between the possible electronic structures, N≡C–S and N=C=S.^32^

K­[SeCN] was not only the first selenocyanate to be synthesized but also the first to be characterized crystallographically.^33^ Like [OCN]^−^ and [SCN]^−^, the selenocyanate anion was found to be linear. A notable difference from its isovalence electronic lighter homologues is that (from interatomic distances) the resonance form N≡C–Se is more predominant than N=C=Se.

As the atomic number of the chalcogen atom increases, the C–Ch bond becomes progressively weaker. While simple compounds containing [OCN]^−^ and [SCN]^−^ are generally air-stable, though often deliquescent, those with the [SeCN]^−^ anion are almost always sensitive to air. Continuing this trend, it is unsurprising that the [TeCN]^−^ anion is the most sensitive among them.

The discovery of the [TeCN]^−^ anion followed a somewhat convoluted path (see Figure 6). Already in 1845, Berzelius observed the formation of a homogeneous mass when potassium cyanide is melted together with tellurium;^25^ however, no experimental evidence is given if the resulting compound was the [TeCN]^−^ anion. From today’s perspective, the formation of K_2_Te_2_ cannot be ruled out under these conditions. Extraction with water at the time led to decomposition under precipitation of tellurium and dissolution of potassium cyanide. In 1925, Birckenbach and Kellermann reported potentiometric measurements of the tellurocyanate anion,^14^ but the experiments could not be repeated successfully.^34^ In another work, Bergström reported a very slow reaction of tellurium with potassium cyanide in liquid ammonia, but the reaction product defied isolation.^35^ In 1968, [NEt_4_]­[TeCN] was isolated as the first manageable tellurocyanate. Its composition was verified by elemental analysis, the presence of the anion determined by vibrational spectroscopy, but the crystals defied X-ray diffraction.^36^

**Figure 6:**
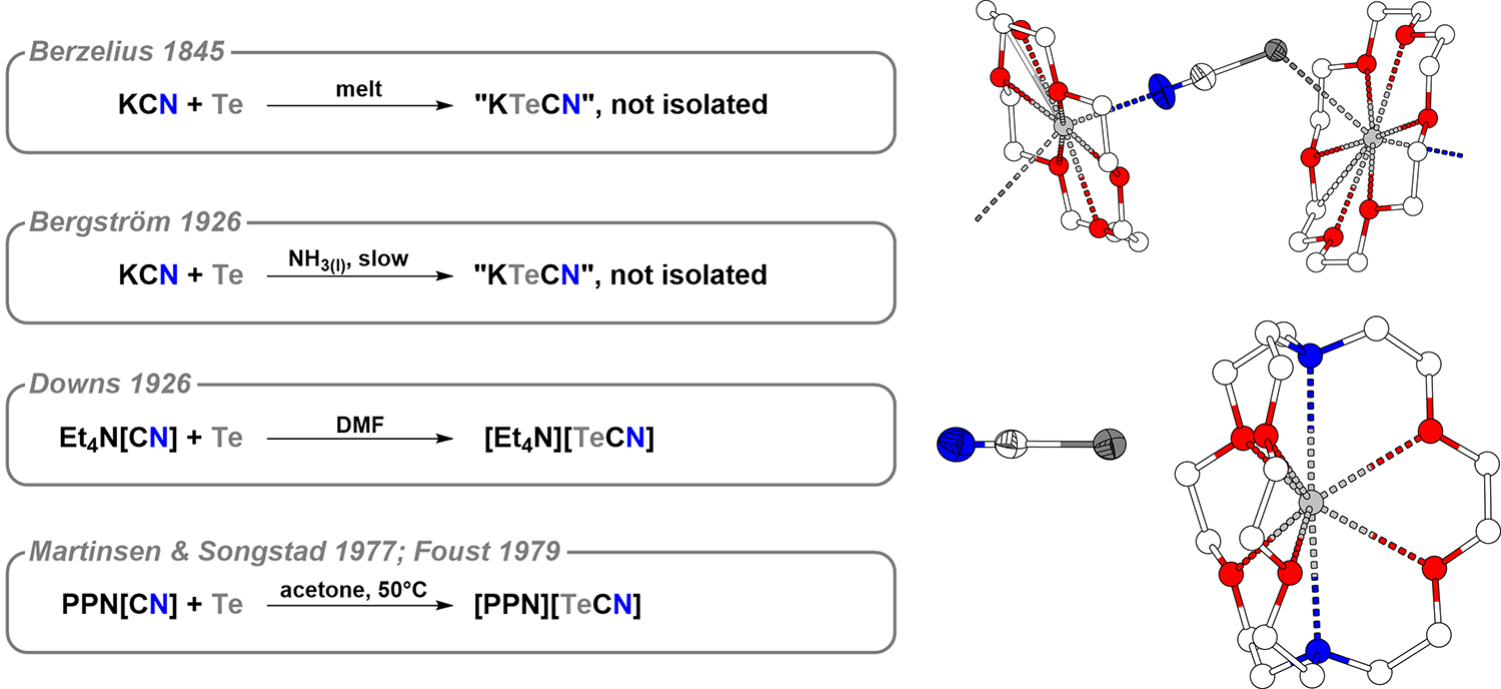
Syntheses of tellurocyanates (left). Crystal structure of [K­(18c6)]­[TeCN] (top) and of [K­(2.2.2-crypt)]­[TeCN] (bottom) (right).

The first unambiguous crystallographic characterization of the [TeCN]^−^ anion was achieved by crystallizing its [PPN]^+^ salt.^37^ The study confirmed that the anion is linear, featuring a long Te–C bond of 2.02(1) Å, supporting the predominance of the N≡C–Te resonance form.[Fn fn9] Notably, all databases include only three structures containing the [TeCN]^−^ moiety: [PPN]­[TeCN],^37^ [K­(18c6)]­[TeCN],^38^ and [K­(2.2.2-crypt)]­[TeCN].^39^

In the first half of the 20th century, X-ray crystallography emerged as a modern analytical method. For the first time, chemists could “see” atoms, albeit indirectly, through diffraction and subsequent model refinement. This powerful tool enabled the unambiguous identification of structural features, ultimately providing definitive proof of the existence of [TeCN]^−^. In the following epoch, we explore how chemists incorporated heavier pnictogen atoms into cyanates and how different strategies led to success.

#### Epoch 3: Modern Methods and Heavy Pnictogen Homologues

It is quite likely that the first synthesis of [OCP]^−^ dates back to 1894 when NaPH_2_ was reacted with CO, but the limitations of that time prevented its characterization (see Figure 7).^40^ This statement is further supported by the fact, that in a similar attempt to originally make NaCP, Grützmacher and co-workers also reported the isolation of Na­[OCP] through the carbonylation of NaPH_2_.^41^ Ninety-eight years after Spanutius, the first undisputed synthesis of [OCP]^−^ was achieved in the form of [(DME)_2_Li]­[OCP].^42^ The synthetic approach used here differed from those used for the nitrogen-containing congeners. Historically, thiocyanates, selenocyanates, and tellurocyanates (and, to some extent, cyanates) were typically synthesized by the simple oxidation of the parent cyanide with elemental chalcogens. The industrial syntheses of cyanates and thiocyanates, on the other hand, start from urea or CS_2_ and are discussed in detail elsewhere.^43–45^ The absence of a free parent cyaphide, such as KCP, renders this synthetic approach unfeasible.^46^ Unlike the typical use of a CN precursor, this method employs a C=O precursor in combination with a phosphorus nucleophile. The reaction begins with [H_2_P]^−^ attacking the carbonyl group of diethyl carbonate in a nucleophilic manner.[Fn fn8] After workup, a [OCP] salt can be isolated either as an alkali metal salt coordinated with dioxane ([Na­(diox)_3_]­[OCP]) or in the form of the corresponding crown- or cryptand-coordinated alkali salts (see Figure 8). However, attempts to strip these substances of coordinating solvents result in decomposition. To date, no solvent-free binary [OCP]^−^ salt has been successfully isolated.

**Figure 7:**
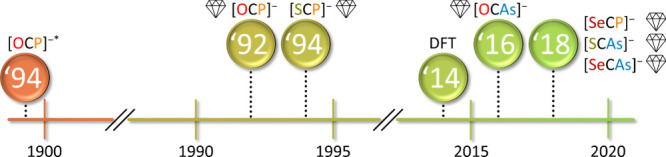
Time line of the seminal syntheses of [OCP]^−^, [SCP]^−^, [SeCP]^−^, [OCAs]^−^, [SCAs]^−^, and [SeCAs]^−^. The first crystal structure determinations are indicated by diamonds. *Limitations of the time precluded the definite proof of its synthesis; however, reactivity is in accordance with [OCP]^−^. For details see Epoch 3: Modern Methods and Heavy Pnictogen Homologues.

**Figure 8:**
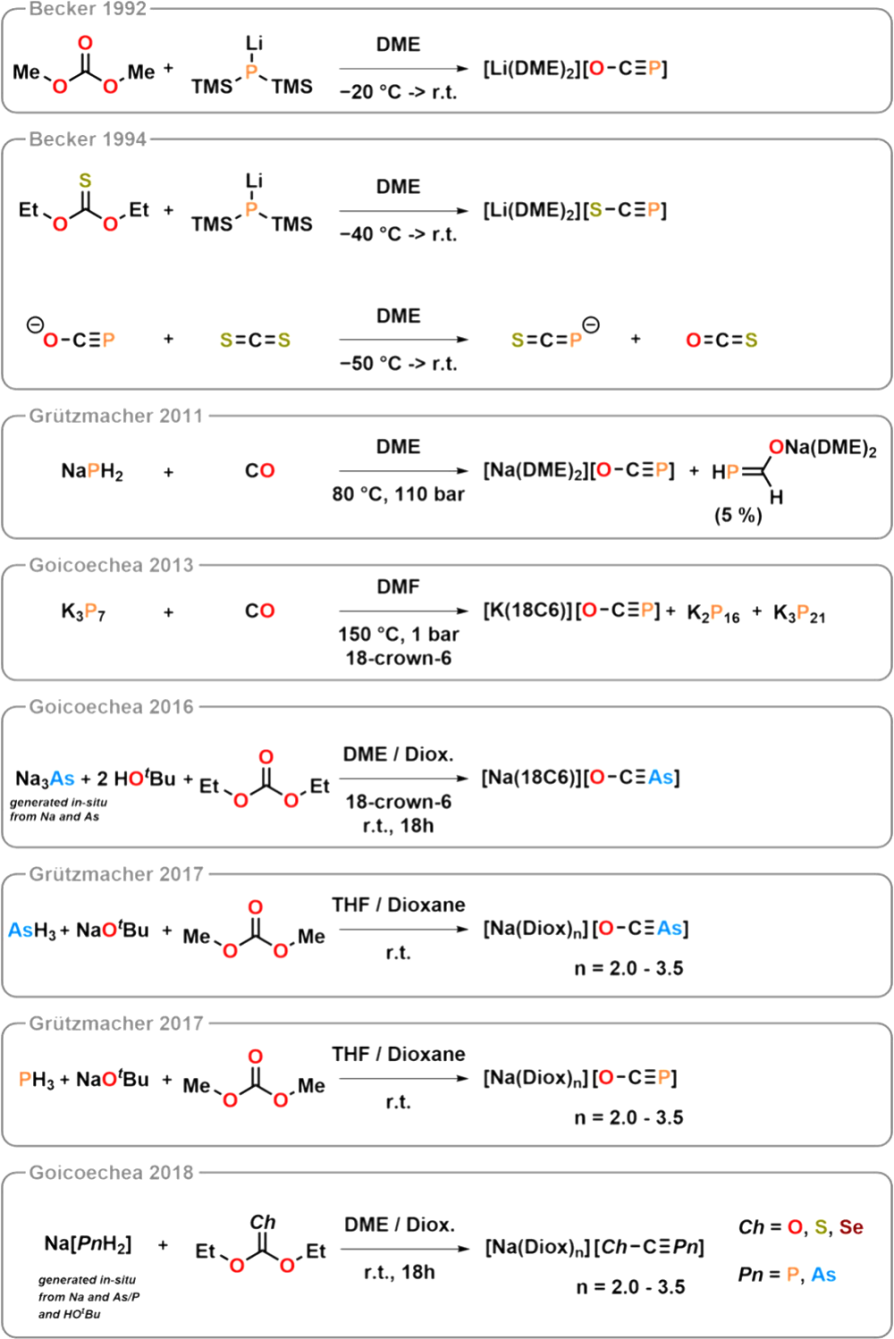
Main synthetic strategies toward the group 15 congeners of the cyanate anion. Further syntheses have been compiled in ref 16.

**Figure 9:**
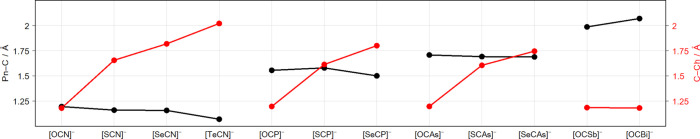
Comparison of the Pn–C (black) and C–Ch (red) bond lengths showing the general trends upon permutation of the elements.

In 1994, Becker isolated the first [SCP]^−^ salt using a similar synthetic strategy, but substituting the organic carbonate with its thionocarbonate counterpart.^47^ It was also demonstrated in this work that a direct exchange of the oxygen atom in [OCP]^−^ with sulfur by reaction with CS_2_ is feasible.^47^ This synthesis was later replicated using sodium as a counterion. The product was coordinated to tungsten (see also below).^48^

As is customary today, the substances were characterized by single-crystal X-ray diffraction. Both [OCP]^−^ and [SCP]^−^ are essentially linear ions that exhibit P–C distances corresponding to triple bonds and C–Ch distances shorter than their single bond length. This hints at a more complex electronic structure, the discussion of which can be found below.

After a report on the computational chemistry suggested that the corresponding arsenic, antimony, and bismuth anions, [OCAs]^−^, [OCSb]^−^, and [OCBi]^−^, respectively, should be thermodynamically stable,^49^ [OCAs]^−^ was synthesized in 2016.^50^ This synthesis employed a modified version of Becker’s original and Grützmacher’s revised protocols. Instead of using [PH_2_]^−^ as the nucleophile, [AsH_2_]^−^ was used, while the remainder of the procedure remained largely unchanged. Due to the poorer overlap of atomic orbitals and the thermodynamically favorable release of CO, the As–C bond is relatively weak. As a result, CO is lost more readily than in [OCP]^−^, making [OCAs]^−^ more sensitive to air than its lighter congener.

Two years later, a general procedure for synthesizing [ChCPn]^−^ anions with Pn = P or As and Ch = S or Se was published, including the seminal syntheses of [SeCP]^−^, [SCAs]^−^, and [SeCAs]^−^.^51^ This method involved substituting the C=Ch building block with either diethyl thionocarbonate or diethyl selenonocarbonate, again with most of the remaining procedure unchanged.

The seminal synthesis of [OCP]^−^ and its revised protocol for facile mass production thereof sparked a “gold rush” in inorganic molecular chemistry.^52^ In just a few years, its reactivity was exploited in many different ways, which is covered by an excellent review.^16^ The reactivity of [OCP]^−^ and [OCAs]^−^ is dominated by the weak Pn–C bond that is in contrast to the sulfur and selenium congeners. Subsequently, their chemistry is still in its infancy, and their known transformations are fully covered in detail below.

Obviously, some cyanate congeners are still missing. Researchers around the world have been searching for, but have yet to discover, [OCSb]^−^ and [OCBi]^−^. Their Pn–C bond is substantially weaker than that in [OCAs]^−^, making their isolation challenging. One could perhaps plan to isolate [SCSb]^−^ first, as the release of CS is less thermodynamically favorable than the release of CO. However, [SbH_2_]^−^ is a very poor nucleophile, and its use in the traditional synthetic protocol was not successful, yet.^53^

[TeCP]^−^ and [TeCAs]^−^ have also not yet been reported. Here, a suitable C=Te precursor is unknown, and telluronocarbonates are notoriously difficult to handle. In fact, the parent tellurocarbonyl difluoride has only been isolated in a solid Ar matrix.^54^ Consequently, a novel synthetic approach is needed.

### Part II: Molecular and Electronic Structures of the Cyanate Anions

All cyanate congeners are essentially linear anions. The linearity is strict if the geometry of the anions is computed in the gas phase or if they reside on an appropriate symmetry element in the crystal structure. Deviations from linearity are likely due to crystal packing effects; however, those deviations are rather small.

In general, several different resonance forms can be formulated for all cyanate congeners, and the most important ones are depicted in the header of Table 1.^55^ Formula A shows a Pn≡C triple bond and a C–Ch single bond, with the negative formal charge on the chalcogen atom. Formula B is the heterocumulene type with double bonds between both Pn and C and C and Ch and the negative formal charge at the pnictogen atom. Structures **A** and **B** generally are most important as there is only one formal charge, and it is located at the most electronegative elements. Formula **C**, in contrast, shows a C≡Ch bond and in total three formal charges, making it less important overall. However, it has some implications regarding reactivity, as discussed below. Finally, formula **D** has only one formal charge; however, that is located on the C atom, and a long bond is formed between the outermost atoms. Other less important formulas have also been calculated and can be found in the literature.^55^

**Table 1:**
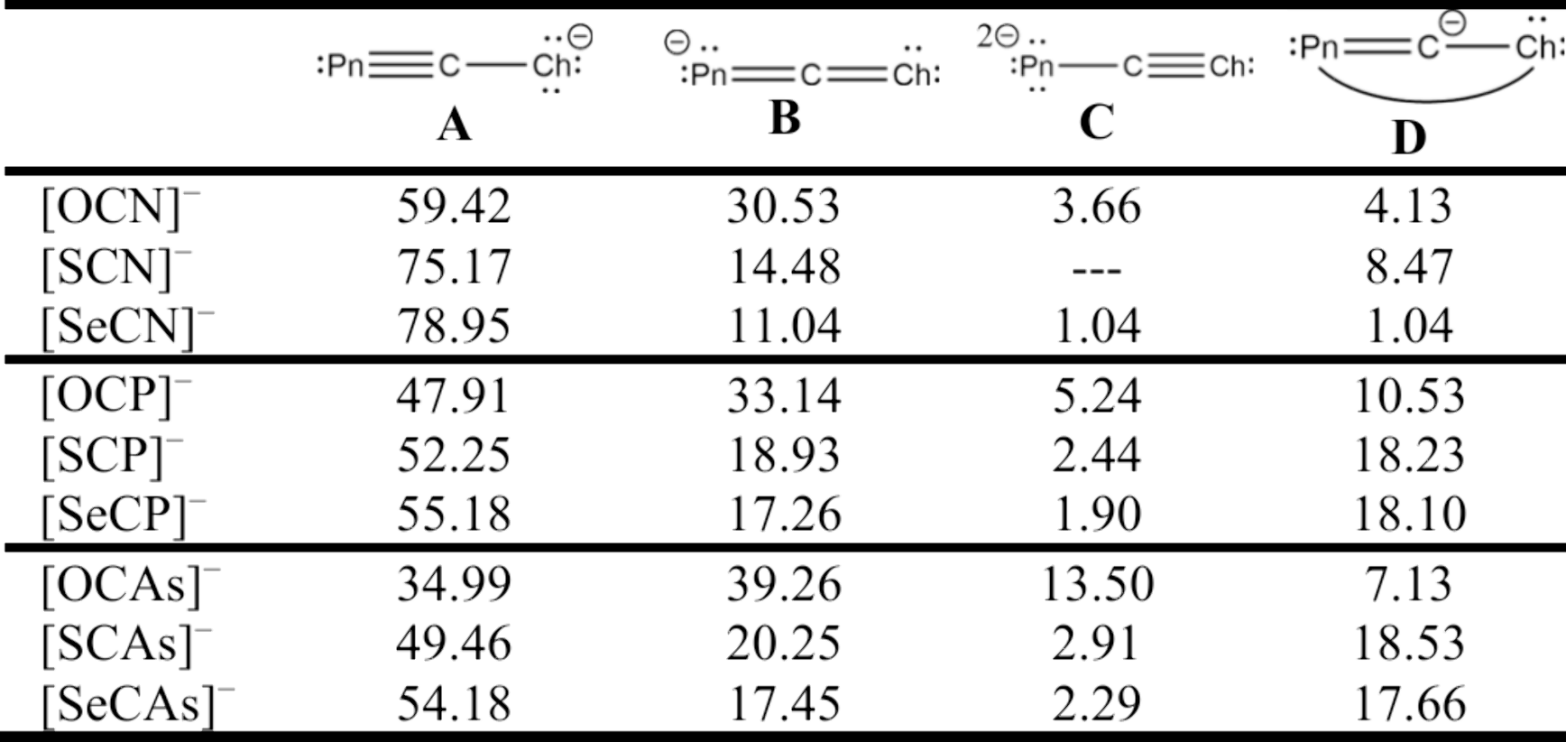
Main Resonance Forms and Their Relative Weights in Percent^55^,^a^

a Values for [TeCN]^−^, [OCSb]^−^, and [OCBi]^−^ have not been reported.

As the period of the Pn and Ch elements increases, the differences in electronegativity decrease and it becomes more important to also discuss resonance forms **C** and **D**.

All bond lengths show distinct trends, as shown in Figure 9. Generally, with an increasing atomic number of the chalcogen, the N–C bond length decreases from 1.195(1) Å^31^ in [OCN]^−^ to only 1.150(6) Å in [K­(crypt-2.2.2)]­[TeCN] and 1.148(1) Å in [K­(18c6)]­[TeCN].^9,37–39^ For [OCN]^−^, this is indicative of significant heterocumulene character (formula **B**, 30.5%) at the expense of resonance form **A**. The N–C bond length is significantly shorter in the respective S, Se, and Te congeners, and the bond is best described as a triple bond (formula **A**, ∼75%) with an increasing Wiberg bond index from O to Te.^39^

Whereas the Pn–C bond is affected by the type of chalcogen in [ChCN]^−^, this does not hold true for the respective phosphorus and arsenic compounds. For [ChCP]^−^, the bond length shows only minor variation around 1.55 Å, with a notable outlier of 1.45 Å in [SCP]^−^, which is probably an artifact of the observed disorder.^51^ Naturally, the Pn–C bond length is even larger in the arsenic compounds, with distances around 1.70 Å.^50^ In both instances, NRT (natural resonance theory) shows an around 50% contribution from resonance form **A**.^55^ Here, an outlier is [OCAs]^−^, which shows only about 35% of the **A** form and a large amount of 39.26% of the heterocumulene. The latter observation can partially be rationalized by the relatively large (13.5%) contribution of form **C**, which exhibits a triple bond between carbon and the chalcogen atom. This form is also important in [OCP]^−^, albeit to a lesser degree (5%). With this “preformed carbon monoxide”, an important aspect of reactivity of [OCP]^−^ and [OCAs]^−^ can be explained. Both can serve as “Pn^–^” synthons under loss of CO, and this strategy has been used to synthesize novel compounds (*vide infra*).^16,56^

Resonance form **D**, with the charge located at the central carbon atom, is virtually unimportant in the [ChCN]^−^ ions but becomes more pronounced especially in the phosphorus and arsenic homologues of the thio- and selenocyanate anions.^55^

All cyanate congeners comprise standard NMR accessible nuclei, all of which are easily detected (literature compiled in footnote [Fn fn10]). Additionally, for the N-, P-, and Se-containing congeners, their respective ^14^N (or ^15^N), ^31^P, and ^77^Se spectra have been reported (see Figure 10). The following trends can be observed. (i) With an increasing Pn atomic number, ^13^C signals are shifted downfield. (ii) In the O → S → Se → Te series, the ^13^C chemical shift is highest for the S congener and lowest for the O congener. (iii) The ^31^P and ^77^Se chemical shifts increase with the atomic weight of Pn. (iv) The ^14^N chemical shift decreases with an increase in the atomic number of Pn.

**Figure 10:**
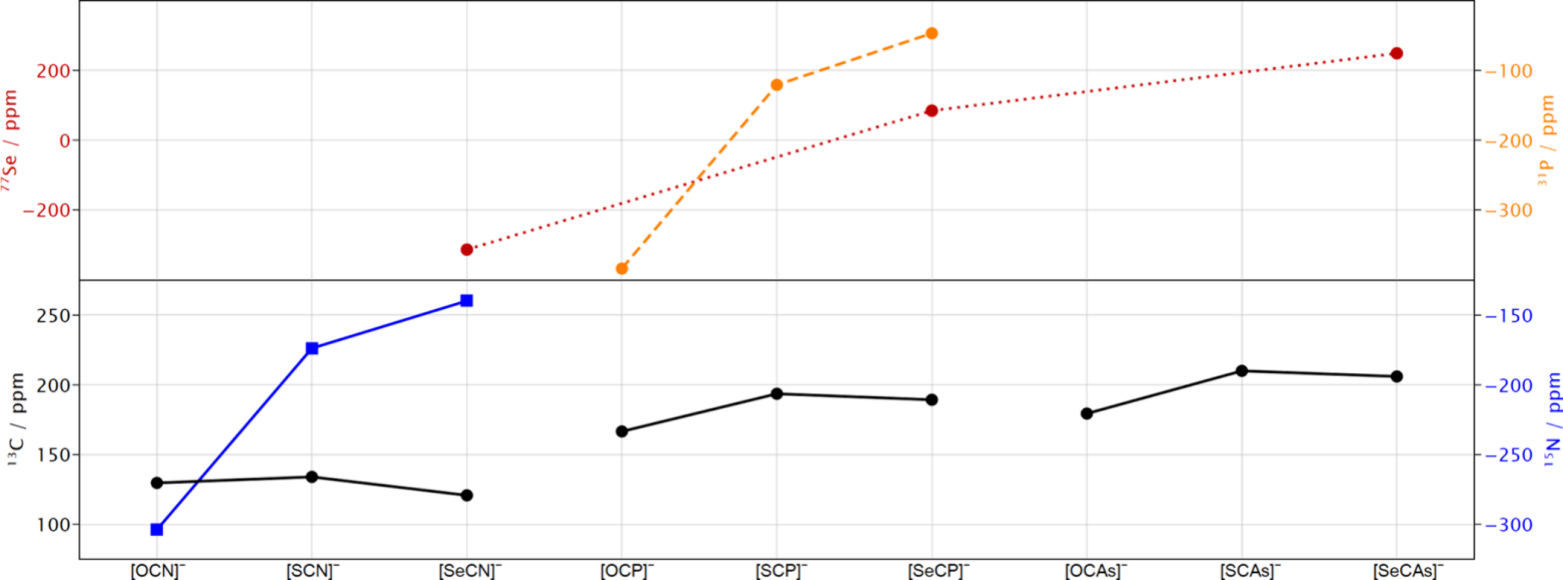
Trends of the ^13^C, ^15^N, ^31^P, and ^77^Se chemical shifts of the cyanate homologues. References are compiled in footnote [Fn fn10].

All of these trends are reflected by the different weights of the resonance formulas (see Table 1),^55^ with the exception of [TeCN]^−^, for which no calculations of this kind have been reported so far. Instead, for [TeCN]^−^, an analysis based on Wiberg bond indices is given, and for NMR analysis, the individual contributions of nonperturbed density, magnetically perturbed density, and each of those split into their scalar and spin–orbit parts have been calculated.^39^

Increasing the atomic number of Pn leads to deshielding of the C atom. Within the series of P and As, resonance **D** is lowest for the O congener, highest for the S congener, and only slightly lower again for the Se congener. The N series breaks this trend, as here resonance **D** is lowest for the Se congener, which again is reflected by the ^13^C chemical shift. It has been shown that the spin–orbit part of the magnetic response density mainly determines the chemical shift for O to Te by its decrease, superimposed by the slight increase of the scalar part, leading to a maximum for S. It is reasonable to assume that this is also true for the P and As congeners; however, these calculations have not yet been reported for those systems.

The ^31^P chemical shift increases from [OCP]^−^ to [SCP]^−^, concomitant with a decrease of resonances **B** and **C** (negative formal charges on P) and the largest difference between [OCP]^−^ and [SCP]^−^. Similarly, the ^77^Se chemical shift increases with an increase in the atomic weight of the Pn atom, which is coincident with a decrease in resonance **A** (negative formal charge on Se) and an increase in resonance **C** (positive formal charge on Se). For ^14^N (^15^N), the trend is a decrease in **B** and **C**, which again is concomitant with a decreased level of shielding.

### Part III: Heavy Cyanate (Coordination) Chemistry

The following paragraphs will mainly deal with the reactivity and coordination chemistry of the arsaethynolate [OCAs]^−^ anion, as well as the coordination chemistry of phospha- and arsaethynthiolate anions [SCP]^−^ and [SCAs]^−^, respectively. Thus, the chemistry of the lighter and parent (thio)­cyanates will not be reviewed here. For those interested in the recent coordination chemistry of these “classical” anions, we refer to the literature ([OCN]^−^,^64^ [SCN]^−^,^65–67^ [SeCN]^−^,^68,69^ and [TeCN]^−^ ^68^). Similarly, the (coordination) chemistry of phosphaethynolate anion [OCP]^−^ will not be summarized here, as there are already excellent recent reviews.^16,70–72^

#### [OCAs]^−^: Reactivity toward Main Group Elements

As already mentioned, arsaethynolate anion [OCAs]^−^ was initially synthesized in 2016,^50^ following a similar approach as for the [OCP]^−^ anion.^52^ Starting from sodium and arsenic, the authors synthesized Na_3_As, which was protonated *in situ* to NaAsH_2_ using *tert*-butanol. Subsequent carbonylation with diethyl carbonate and workup formed [Na­(18c6)]­[OCAs]. Alternatively, bubbling AsH_3_ through a solution of NaO^t^Bu in the presence of dimethyl carbonate also gives facile and scalable access to the arsaethynolate anion (see Figure 8).^73^ The anion is highly susceptible to oxidation, decomposing to either As_7_^3–^ or elemental arsenic, when exposed to air or mild oxidants. With organic substrates, such as ketenes or carbodiimides, selective [2+2] cycloaddition is observed, forming [As­(C­(O))_2_CPh_2_]^−^ **1** or [AsC­(O)­(CNDipp)­NDipp]^−^ **2**, respectively (Figure 11). Similar [2+2] cycloaddition reactivity has also been reported for [OCP]^−^.^74^ On the contrary, reaction with diisopropylphenyl isocyanate (DippOCN) forms [As­(C­(O))_2_(NDipp)_2_]^−^ **3**, in a less selective reaction. This can be explained by the reaction between the [OCAs]^−^ anion and 2 equiv of DippOCN by the extrusion of 1 equiv of carbon monoxide. Byproducts of the reaction were identified as As_10_^2–^ and As_12_^4–^ (in both *C*_2*h*_ and *D*_4*h*_ symmetry). A similar five-membered heterocycle has also been observed in the reaction between DippOCN and [OCP]^−^.^75^

**Figure 11:**
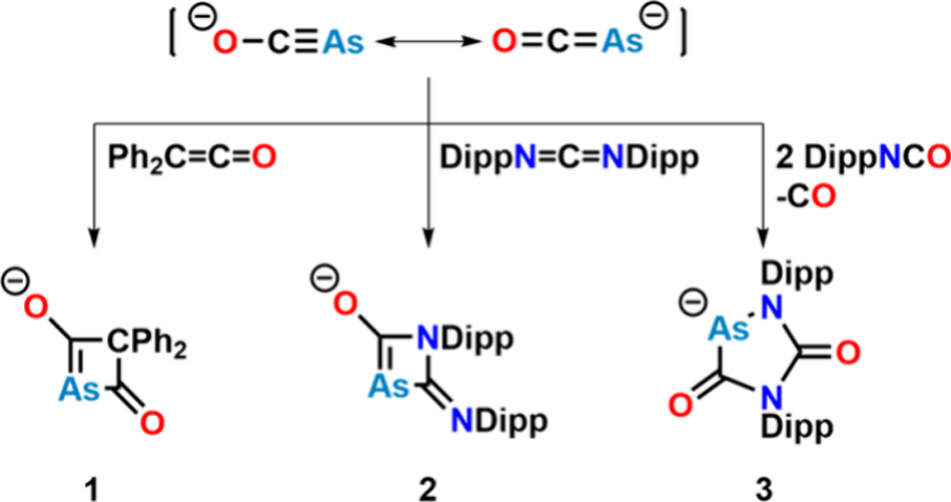
[2+2] cycloaddition reactivity of the arsaethynolate anion and reactivity toward DippNCO forming new (anionic) arsenic-functionalized heterocycles.

Studying the potential of the [OCAs]^−^ anion to act as an arsenide source, its reactivity toward bulky stannylenes was studied (Figure 12).^56^ Reaction between bis­(terphenyl)­stannylene **4** ^76^ and [Na­(18c6)]­[OCAs] over 2 weeks resulted in the formation of cluster **5**.^56^ Computational investigations revealed that the cluster formed via arsaketenyl complex **6**, which photodegrades under ambient conditions to arsastannylene **7**. Both species can be isolated and structurally characterized by performing the reaction at very low temperatures. Notably, crystals of **6** photodecompose on the diffractometer, so crystals of **6** always contain **7** as an impurity. Rearrangement of a terphenyl substituent in **7** results in the formation of **8**, which undergoes a [2+2] cycloaddition reaction with itself, forming cluster **9**. Finally, elimination of 1 equiv of terphenyl leads to the observed formation of cluster **5**.

**Figure 12:**
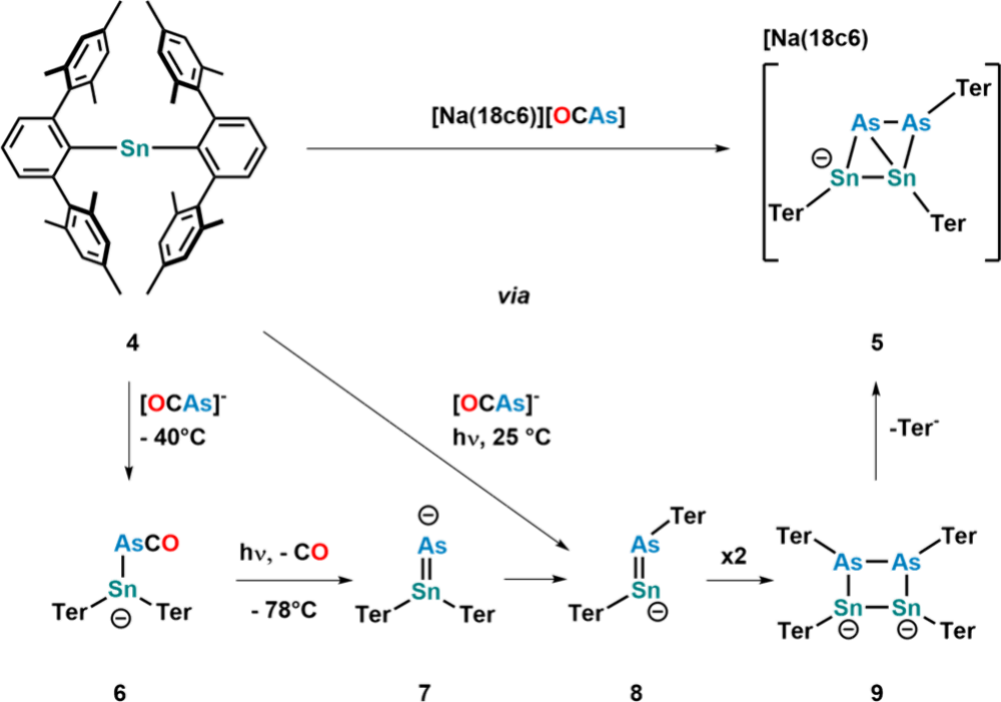
Reactivity between the [OCAs]^−^ anion and a sterically encumbered stannylene to yield cluster **5**. The bottom panel shows the computed mechanism, including the conditions to synthesize the mechanistic intermediates independently. The [Na­(18c6)]^+^ counterion has been omitted for the sake of clarity in the mechanism.

Changing the stannylene source from (Ter)_2_Sn (Ter = 2,6-dimesityl-phenyl) to the halide containing tin source (Ter)­SnCl **10** led to the isolation of heterocubane [TerSnAs]_4_ **11** forming via a putative “terphenylSnAs” intermediate. If the reaction is carried out with [OCP]^−^, no similar heterocubane formation is observed. Theoretical calculations, however, suggest that this is due to kinetic rather than thermodynamic reasons, as the formation of a heterocubane is highly exothermic for both P and As. Furthermore, the study shows that the terphenyl ligand is too small to stabilize the proposed arsastannyne intermediate (Figure 13).

**Figure 13:**
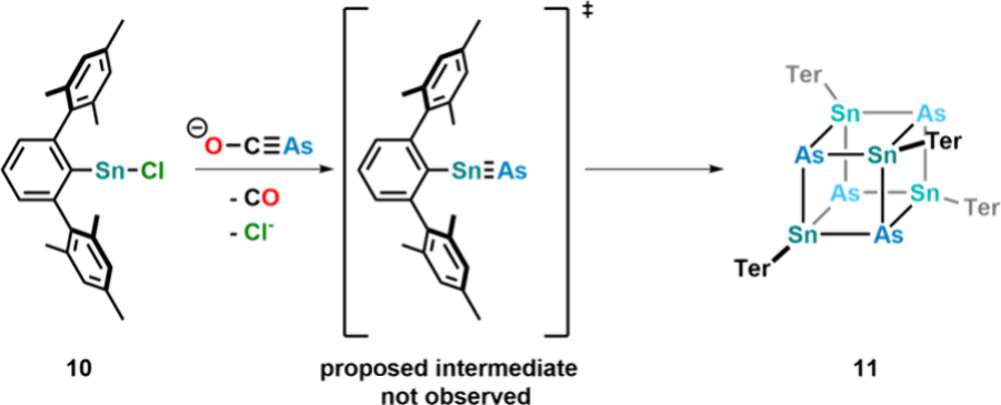
Formation of a tin–arsenic heterocubane via a proposed arsastannyne intermediate.

Many efforts have been made to isolate terminal group 14 arsinidene complexes, however, with no success. In 2017, the synthesis of substituted germylidenylarsindenes was reported (Figure 14).^73^ The synthesis was achieved by the reaction between (BDI)­GeCl **12** (BDI = β-diketiminate) and Na­[OCAs] to give arsaketenyl complex **13**. In the presence of NHC or triphenylphosphine, this complex extrudes carbon monoxide, giving facile access to “capped” germylidenylarsinidenes **16** and **17**, respectively. The authors further mentioned that capping of the arsinidene is crucial since photolysis under ambient light commences. In the absence of trapping reagents, this led to “head-to-tail” dimerization of intermediately formed arsagermyne **14**, forming 1,3-digerma-2,4-diarsacyclobutadiene complex **15**.

**Figure 14:**
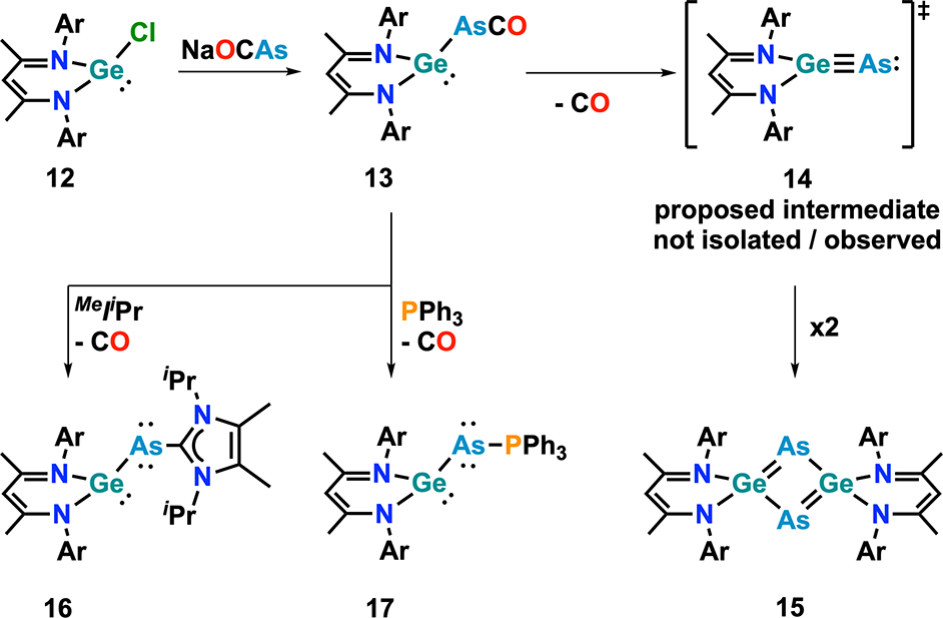
Synthesis of BDI-supported NHC and PPh_3_-capped germylidenylarsinidenes via CO extrusion from an arsaketenyl complex. Ar = diisopropylphenyl (Dipp).

Further studying the reactivity of germanium, trimethylphosphine-stabilized germylidenylarsinidene **20-As** was synthesized (Figure 15) using sterically highly encumbering hydrinacene ligands (^Ms^Fluind^^t^Bu^)^77^ starting from Ge­(II)­Cl complex **19** and Na­[OCAs] in the presence of excess PMe_3_. The authors proposed that the reaction proceeds via the intermediate formation of arsaketenyl complex **21** similar to previous reports (*vide supra*).^73^ However, without PMe_3_ no arseketenyl complex was isolated.^77^ Similarly, germylidenylphosphinidene **20-P** was isolated from the reaction between **19** and Na­[OCP] in the presence of PMe_3_. Although theoretical calculations proposed similar electronic structures of **20-P** and **20-As**, they showed large differences in their reactivity. This was examined by studying their cycloaddition chemistry using differently substituted alkynes (TMS-alkyne and 4-*tert*-butylphenylalkyne). While the reaction of both these alkynes with **20-P** afforded differently substituted phosphagermabenzen-1-ylidenes **22** and **23**, the use of **20-As** gave access to either arsagermene complex **24** or arsolylgermylene complex **25**.^77^

**Figure 15:**
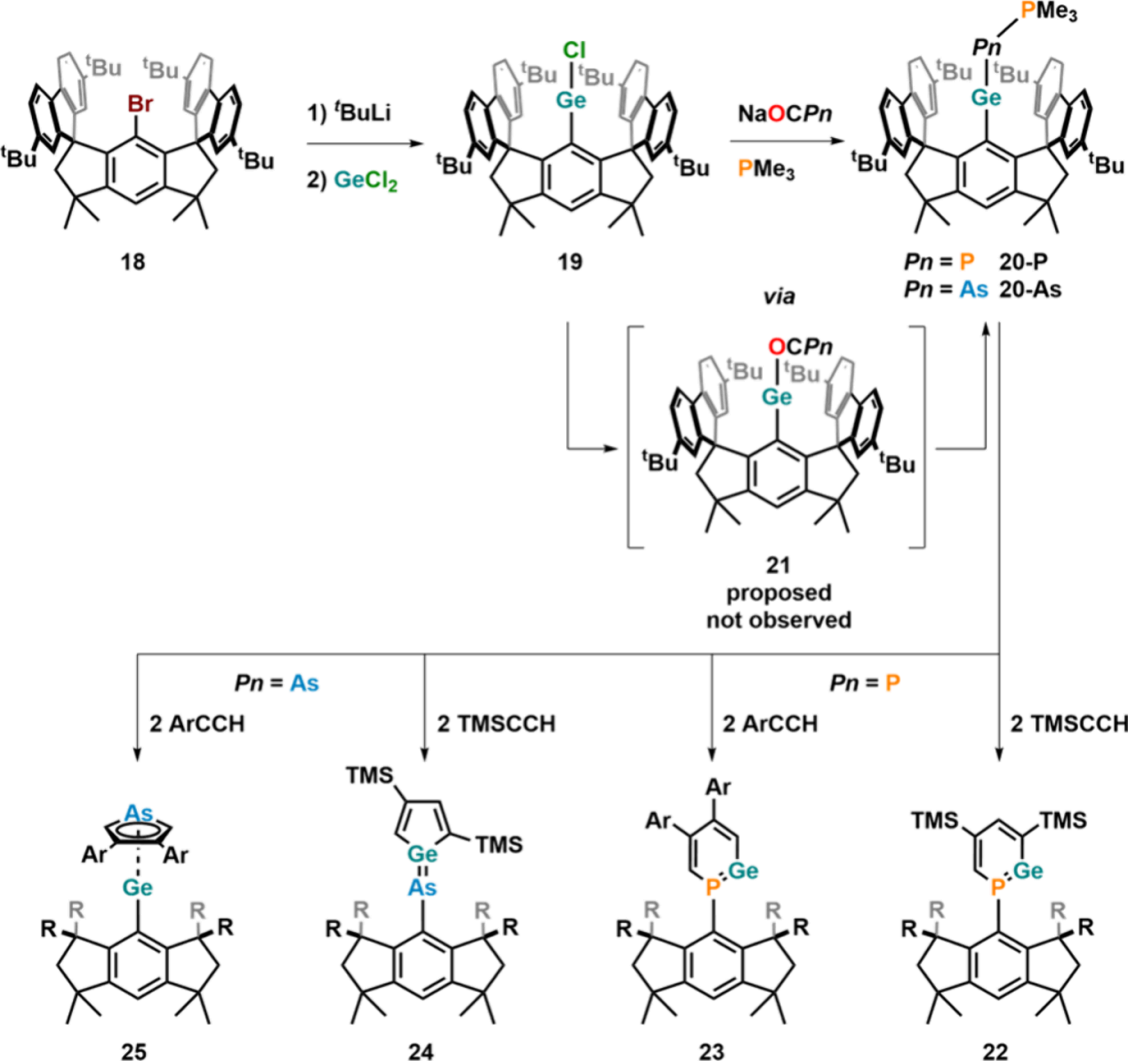
Hydrindacine-supported germylidenylpnictinides (P and As) and their cycloaddition chemistry. Ar = 4-^t^Bu-C_6_H_4_.

Shifting focus to lighter tetrel elements, the synthesis of silylene–arsinidene complexes stabilized by a zinc­(II) fragment was explored (Figure 16).^78^ Starting from -*ate* complex **26**, the authors synthesized arsaketenyl complex **27**, which was not stable in solution or the solid state for prolonged times. Coordination of ^Me2^I^i^Pr to the zinc center facilitated LiCl elimination, leading to neutral zinc complex **28**, which underwent clean metalation with Na­[OCAs] to form NHC-stabilized arsaketenyl complex **29**. Addition of an N-heterocyclic silylene (^^t^Bu^NHSi) induced decarbonylation of the arsaketenyl unit, giving access to zinco arsinidene–silylene complex **30**. The NHC ligand is transferred from Zn to Si. Attempts to prepare an NHC-free version of this complex failed, and reaction of *in situ*-generated **27** with ^^t^Bu^NHSi gave access to only dimeric complex **31** with a Si_2_As_2_ core unit. Notably, the addition of ^Me2^I^i^Pr to this complex gave access to **30** again, splitting the Si_2_As_2_ core. Given the fact that steric effects seem to play a non-negligible role for the formation of monomeric or dimeric complexes, the authors further reacted *in situ*-generated **27** with the more bulky silylene ^Dipp^NHSi (^Ar^NHSI). Indeed, this reaction gave access to monomeric silylene–arsinidene complex **32**, without any further ligands being coordinated to the Si atom. Further exploring the reactivity of unsubstituted silylene–arsinidene **32**,^79^ the group probed its reactivity toward water and ammonia. This resulted in cleavage of the X–H bond (X = OH or NH_2_) and the addition of water or ammonia across the Si=As bond (**33-**X). Similar reactivities were also observed in related silaphosphenes.^80,81^ Turning to mild oxidants, the reaction of **32** with nitrous oxide afforded diarsene complex **34** with a [As_2_]^2–^ unit, bridging two zinco siloxy moieties. The Si=As bond was fully cleaved in this process, and the metals were redistributed. The authors assume that upon N_2_O exposure, monooxygenation at the As=Si bond occurs, leading to migration of Zn to the oxygen atom producing zinco siloxy­(arsinidene) [(BDI)­ZnO­(^Dipp^NHSi)­As], which dimerizes to give the final product. Oxidation with carbon monoxide gave access to complex **35** with an arsaethynolato moiety after crystallization. This is surprising because if oxidation proceeds via the above proposed zinco siloxy­(arsinidene), regioisomers **35′** would have been the expected reaction product. However, if the product is examined in the liquid phase, a mixture of compounds **35** and **35′** is present, indicating an isomerization equilibrium between these two compounds in solution. Theoretical calculations indicate an energy difference of only 1 kcal mol^–1^ between the two isomers.

**Figure 16:**
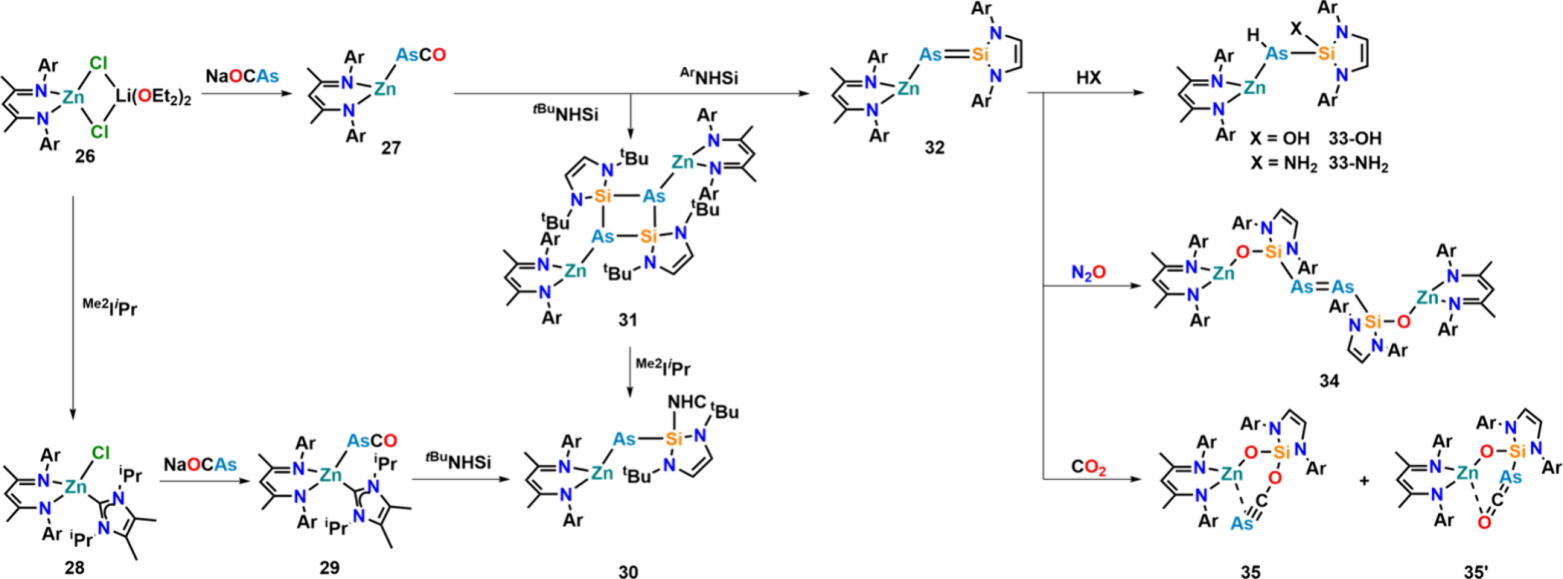
Synthesis and reactivity of silylene–arsinidenes toward N-heterocyclic carbenes, water, ammonia, nitrous oxide, and carbon dioxide. Ar = 2,6-diisopropylphenyl (Dipp).

Focusing on the lightest group 14 element, carbon, the synthesis of NHC-stabilized arsinidenes **36** (Figure 17) was reported.^82^ These are accessible either by TMS-F extrusion starting from a 2,2-difluoroimidazole **37** and tris-trimethylsilyl-arsine, followed by methanolysis of the remaining As-TMS bond in **38** or by direct reaction between the corresponding imidazolium salts **39** and Na­[OCAs]. These ligands resemble the corresponding NHC phosphinidenes, and their coordination chemistry toward main group^83–85^ and transition metals^86^ is currently under investigation.

**Figure 17:**
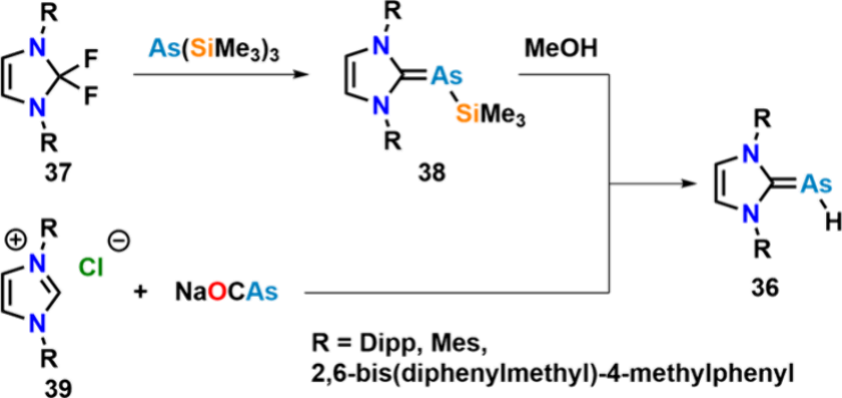
Synthesis of NHC–arsinidenes through TMS-F elimination from As­(TMS)_3_ or through CO extrusion from Na­[OCAs].

Moving from carbon to phosphorus-based electrophiles, the first free phosphinidene **42** was synthesized through the reaction between chlorodiazaphospholidine **40** and Na­[OCP], followed by photolysis of intermediate phosphaketene **41**.^87^ Switching the cyanate source to Na­[OCAs] was envisioned to give access to the terminal arsinidene (Figure 18).^88^ However, repeating the reaction using similar conditions did not give access to a putative arsaketene but, depending on the [OCAs]^−^ source, resulted in the formation of either **43** or **44** under concomitant CO release, implying that the arsaketene might be thermally unstable. Starting from [Na­(18c6)]­[OCAs], **43** formed via a putative phosphine–arsinidene, undergoing a [2+2] cycloaddition reaction with free [OCAs]^−^. When the encapsulating crown ether was removed and starting from [Na­(diox)_3_]­[OCAs], a complex mixture was observed, from which **44** was isolated as a crystalline material. Halide abstraction (with BArF_24_) from chlorodiazaphospholidine **40** led to an intermediate phosphenium salt. The subsequent reaction with [Na­(diox)_3_]­[OCAs] gave clean access to bicyclic tetraarsine compound **45**. Since isolation of the free phosphino–arsinidene was not possible, the group focused on trapping the intermediate phosphine–arsinidene. The first choice was the use of isonitriles, since they are known to quickly react with carbenoids, such as nitrenes^89,90^ or free phosphino–phosphinidene **42**.^87,91^ Although NMR monitoring experiments showed the formation of a putative (Ar*NCH_2_)_2_PAs­(CNR) species, this was only a byproduct. Given the large excess of nitrile that needed to be used to suppress formation of **44** and **45**, the major product of the reaction was “spirocyclic” compound **46**, in which no P–As bond is present. This also hampered the identification of a transient phosphino–arsinidene in the reaction. Changing the trapping reagent to cyclic alkyl amino carbenes (cAACs) or N-heterocyclic carbenes (NHCs) gave access to arsa–allenyl species **47** and NHC-trapped phosphino–arsinidene **48**, respectively. The former structurally resembles Escudié’s arsallene.^92^ Given the success of NHCs to trap the phosphino–arsinidene, the authors also tested PPh_3_ as a trapping reagent, and indeed, phosphine-trapped phosphino–arsinidene **49** was isolated. This species also acted as a phosphino–arsinidene transfer reagent. Upon reaction with low-oxidation state transition metals such as W­(CO)_3_(PrCN)_3_, the reaction gave access to three new compounds: bicyclic tetraarsenide **45**, W­(CO)_3_(PrCN)_2_(PPh_3_), and arsenide complex **50** with a nucleophilic arsinidene fragment. The formation of the latter distinguished the phosphino–arsinidene from the phosphino–phosphinidene, which typically displays electrophilic reactivity,^91^ and the isolation of a related tungsten complex of phosphino–phosphinidene **42** was not possible.

**Figure 18:**
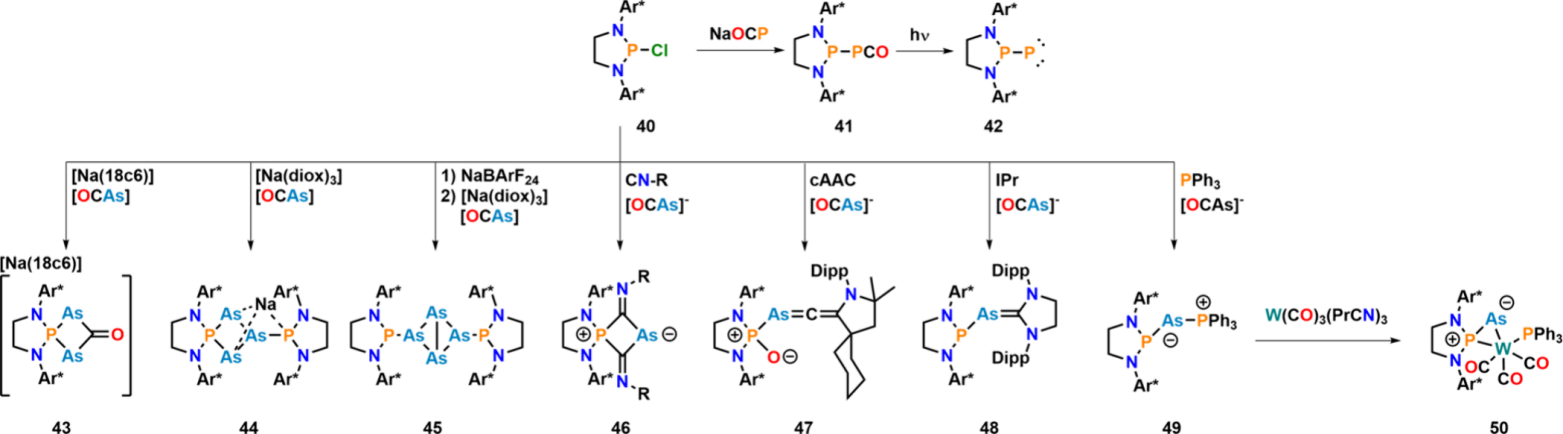
Synthesis of a free phosphino–phosphinidene and attempted synthesis of an analogous phosphino–arsinidene, including its interception reactions. Ar* = 2,6-bis­[(4-*tert*-butylphenyl)­methyl]-4-methylphenyl.

#### [OCAs]^−^: Reactivity toward Transition Metals

The potential of the heavy cyanates to act as pnictogen atom transfer reagents is of course not limited to main group elements, and a large variety of transition metal reactivity of the [OCAs]^−^ anion has also been reported. For example, taking up the strategy to trap terminal pnictinides with isocyanides, terminal phosphide and arside complexes of titanium­(IV) were captured, yielding new cyanophosphide (**52-P**) and cyanoarsenide (**52-As**) moieties (Figure 19).^93^ That synthetic strategy employed the use of an isonitrile-coordinated Ti^II^ species (**51-Ti**), which readily reacted with Na­[OCP] and Na­[OCAs], to form the corresponding complexes. The oxidation state of the titanium center in this process is either Ti^II^ with an [Ad-NCPn]^−^ or Ti^IV^ with an [Ad-NCPn]^3–^ ligand. Bond analysis and theoretical calculations (two fragment effective oxidation state analysis) indicated a Ti^II^ oxidation state to be slightly favored over Ti^IV^. For [OCP]^−^, the reaction was clean, while for [OCAs]^−^, large amounts of (partially unknown) side products were observed, resulting in very low yields of **52-As**. Given the masked Ti^II^ configuration in **52-P**, the compound reacted cleanly with non-oxidizing nucleophiles such as AlMe_3_ to afford P-bridged complex **53**, while the use of platinum chloride yielded Ti^III^ complex **54**. Interestingly, this reactivity was limited to titanium as similar vanadium­(II) complexes (**51-V**) did not yield corresponding vanadium complexes but only coordinate [OCP]^−^ in a κ^1^-O fashion (**55**). This is in line with previous reports by the same group showing that the [OCP]^−^ anion is activated by Ti^III^ ^94^ but not by isostructural V^III^ complexes.^95^

**Figure 19:**
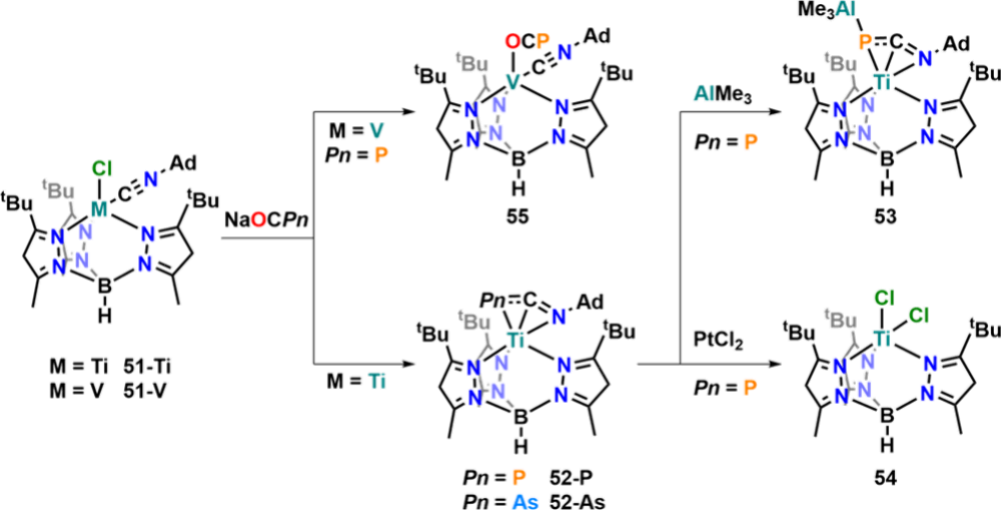
Synthesis of cyanophosphide and cyanoarsenide Ti^II^ complexes via trapping isonitrile and trapping of titanium pnictides.

Further exploring the titanium­(II) chemistry with the arsaethynolate anion, the reaction between **56** and Na­[OCAs] in THF/toluene mixtures resulted in the formation of dimeric Na/K-bridged complex **57** (Figure 20).^96^ In contrast, in pure THF, the formation of monomeric complex **58** as a discrete salt was observed. Both complexes were protonated using a variety of phenols but did not react with amines such as pyrrole or diphenyl aniline, rendering the p*K*_a_ of the arsenide ligand to be between 18 and 23. Phenolic protonation of **57** or **58** resulted in the formation of parent arsinidene complex **59**, with a Ti=AsH bond distance of 2.3775(4) Å being notably longer compared to the Ti≡As in **57** and **58** (2.2661(5)–2.2857(10) Å) but also shorter compared to the Ti arsinidene (Cp_2_Ti = AsAr′(PMe_3_)) (Ar′ = 2,6-{2,6-^i^Pr_2_C_6_H_3_}­C_6_H_3_; 2.4726(8) Å).^97^ Deprotonation of arsinidene complex **59** with benzyl potassium yielded K-bridged dimeric complex **30**, which upon potassium encapsulation (2.2.2-crypt) converted into **58**.

**Figure 20:**
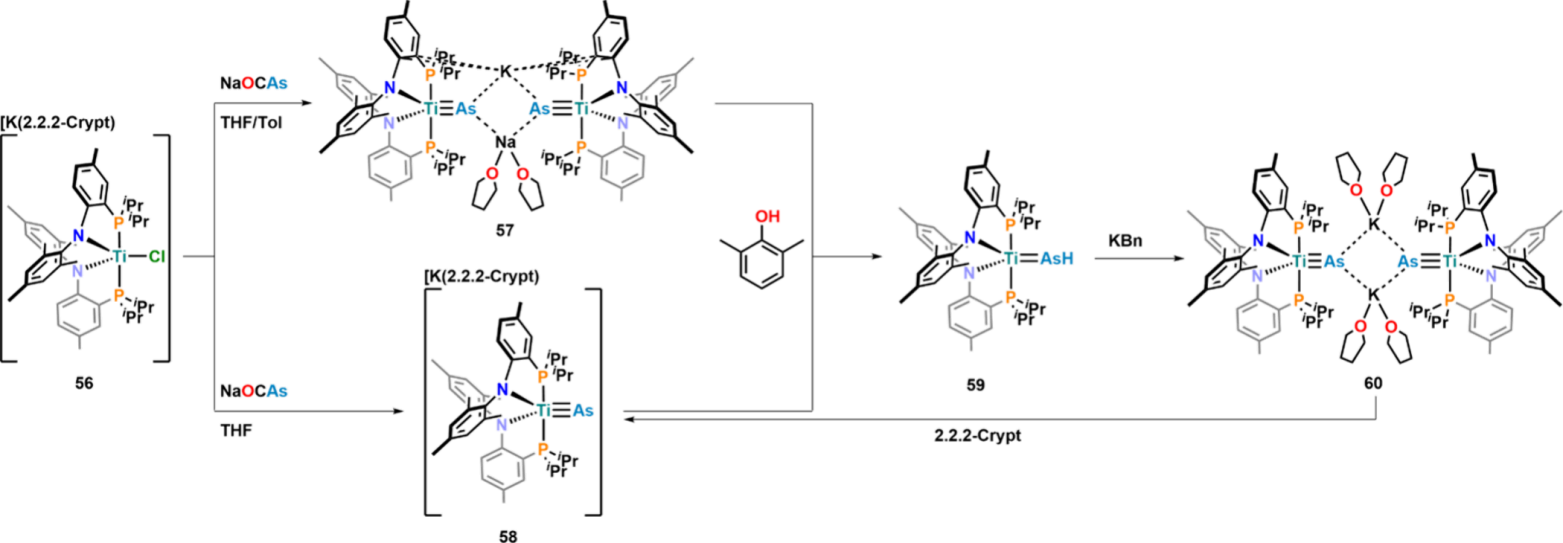
Synthetic strategies to the first Ti arsenide complexes and their parent arsinidene complex.

Turning to group VI chemistry, the synthesis of anionic tungsten arsenide complex **61-As** ^98^ from the reaction of [OCAs]^−^ and the W­(IV) complex W­(ODipp)_4_ was reported (Figure 21).^99,100^ Notably, the decarbonylation is not limited to the arsaethynolate anion, but the phosphaethynolate and even the parent cyanate anion react similarly with W­(ODipp)_4_ to give access to isostructural pnictide complexes **61-P** and **61-N**.

**Figure 21:**
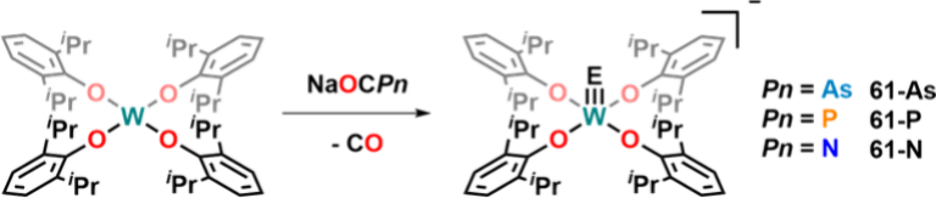
Reductive decarbonylation of (heavy) cyanates by W­(ODipp)_4_, yielding the corresponding tungsten pnictide complexes.

A similar mode of reactivity was reported in 2018, starting from low-valent rhenium­(III) complex **62** (Figure 22).^101^ The reaction of this complex with Na­[OCP] or Na­[OCAs] forged the corresponding rhenium­(V) phosphido (**63-P**) and arsenido complex (**63-As**) with a d^2^ metal center. Interestingly, upon one-electron oxidation, pnictide–pnictide coupling occurred and diphosphide/diarsenide complexes **64-P** and **64-As** were obtained as the major products. The mechanism of E–E coupling is somewhat unclear and involves the loss of [Re­(PyrPz)­(PNP)]^+^, which cannot be independently synthesized. EPR experiments at −80 °C showed only weak signals, indicating a very fast E–E bond formation mechanism. NBO analysis revealed double bond character, and the Re–Pn=Pn–N units are best described as [P_2_]^0^ and [As_2_]^0^.

**Figure 22:**
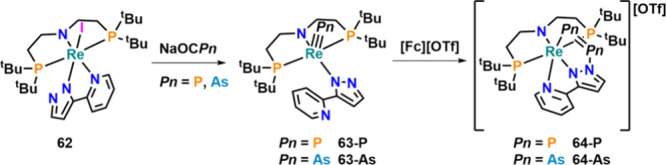
Synthesis of terminal rhenium­(V) pnictide complexes and Pn–Pn coupling reaction upon one-electron oxidation.

Turning to the later transition metals, following their seminal work on platinum triplet nitrenes,^102–104^ Schneider and co-workers expanded their efforts in synthesizing terminal triplet pnictinidenes of palladium and platinum (Figure 23).^105^ Starting from triflate complexes **65-Pd** and **65-Pt**, salt metathesis reactions with Na­[OCP] or Na­[OCAs] afforded phospha- and arsaethynolate complexes **66-M** and **67-M** (M = Pd and Pt), respectively. Irradiation of these complexes with 456–525 nm for 90–180 h afforded dipnictinide complexes **70-M** and **71-M** with new P–P and As–As bridging ligands, respectively, which can best be described as [Pn_2_]^2–^ units. In the case of the phosphorus and arsenic bridges, they can be further oxidized to radical [P_2_]^−^ and [As_2_]^−^ bridges in **72** and **73**, respectively. Notably, the diphosphanide bridge in **72** can also be further oxidized to a [P_2_]^0^ bridging fragments.^106^ In crystallo irradiations furthermore afforded terminal triplet arseninidene complexes **68-M** and **69-M** with Pt–P and Pt–As distances of 2.25(4) and 2.36(3) Å, respectively.^105^ Notably, the authors also report the synthesis of the remaining [Pn_2_]^2–^ bridges of antimony and bismuth but not starting from the (yet unknown) cyanate precursors.

**Figure 23:**
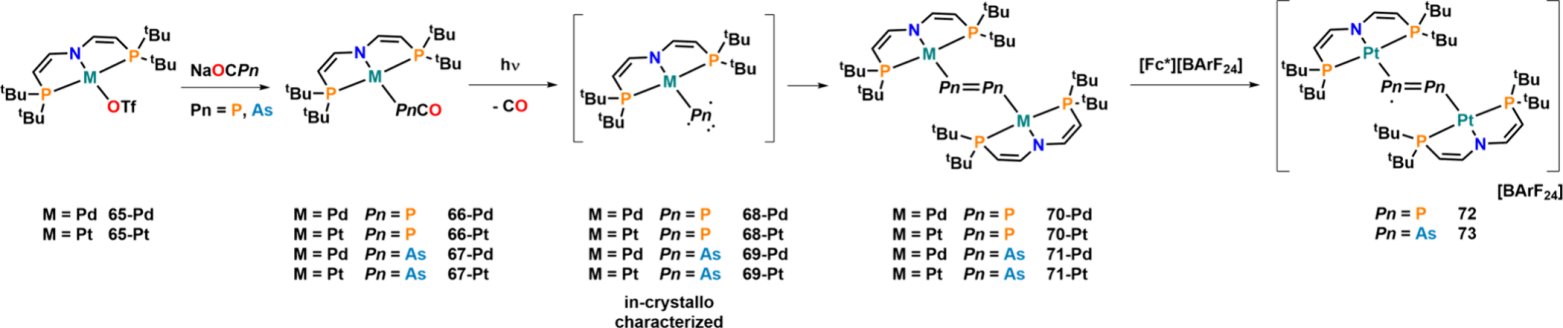
Synthesis of palladium and platinum dipnictinides via light-induced decarbonylation of phospha- and arsaketenyl Pd/Pt complexes via transient triplet pnictinidene complexes.

Using low-valent Ni­(I) complex **74**, the decabonylative reduction of [OCAs]^−^ was reported in 2018 (Figure 24).^107^ Instead of the formation of a terminal arsenide, μ^2^:η^2^,η^2^-As_2_ complex **75-As** was observed as the major product of the reaction. Similarly, starting from [OCP]^−^, μ^2^:η^2^,η^2^-P_2_ complex **75-P** was obtained. Mechanistic studies using only 0.5 equiv of Na­[OCPn] (Pn = P or As) revealed that Pn–Pn bond formation in **75-P** and **75-As** worked via initial side-on coordination of [OCPn]^−^, bridging two nickel­(I) units (**76**), followed by subsequent Pn–CO splitting, forming NHC–phosphinidene and NHC–arsinidene complexes **77-P** and **77-As**, respectively. These reacted with another 0.5 equiv of [OCAs]^−^ or [OCP]^−^ forming the final product **75-P** or **75-As**, respectively. Furthermore, the mixed As/P product was obtained by the stepwise addition of 0.5 equiv of Na­[OCP] and Na­[OCAs]. If **75-P** and **75-As** were treated with carbon monoxide (2 bar), they released the pnictogen atoms. While in the case of arsenic complex **75-As** this led to the direct precipitation of gray arsenic, for phosphorus complex **75-P**, a transient P_2_ molecule was trapped through the addition of 2,3-dimethyl-1,3-butadiene.

**Figure 24:**
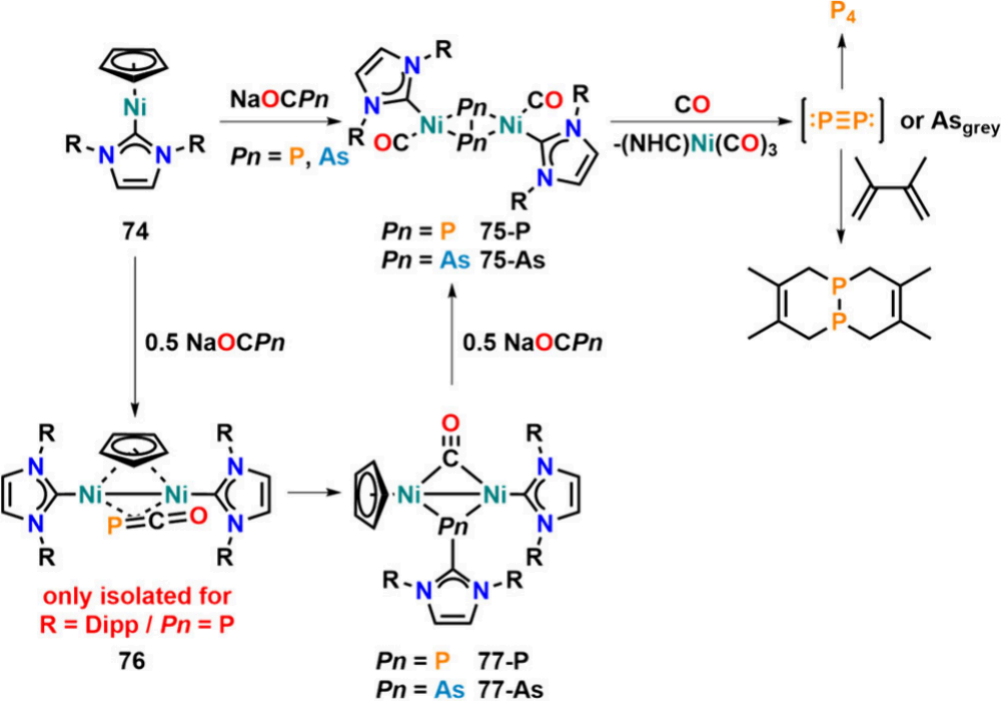
Reactivity of low-valent Ni–NHC complexes toward [OCP]^−^ and [OCAs]^−^ and P atom transfer or gray arsenic formation from the resulting complexes.

Starting from simple NiBr_2_(THF)_1.5_, salt metathesis with Na­[OCP] and Na­[OCAs] cleanly afforded amorphous nickel pnictides NiP and NiAs, respectively. Formation of this species was proven by EDX spectroscopy (among others), and the authors claim that the materials form via the transient formation of BrNi­(OCPn) (Pn = P or As), subsequent E–CO bond splitting, and elimination of oxalyl bromide to yield the nickel pnictides. These are excellent starting materials to form γ-NiOOH_*x*_, which can be used for electrocatalytic water oxidations.^108^

#### [OCAs]^−^: Reactivity toward f Elements

Surprisingly, no lanthanide chemistry with [OCAs]^−^ has been described, yet; also, the phosphaethynolate anion has scarcely been coordinated to the lanthanides yet.^109,110^ So far, only one report in which the [OCAs] anion has been coordinated toward scandium and yttrium has been reported, in which the anions display the expected κ^1^-O coordination mode.^111^ On the contrary, within the 5f elements two reports of arsaethynolate coordination toward uranium­(III) and uranium­(IV) are present. In 2018, the activation of [OCAs]^−^ at a uranium­(III) tris-phenolate framework **78** (Figure 25) was reported. Contrasting the previous activation modes, the uranium complexes did not induce decarbonylation of the [OCAs]^−^ moiety but reductively split the O–CAs bond yielding a μ-oxo diuranium complex **79**, with a scarcely observed cyarside ligand.^112^ A similar activation has been seen for the phosphaethynolate anion on uranium.^113^ The complex reacted with another 1 equiv of Na­[OCAs] to form 1,3-diarsallenide-bridged diuranium­(V) complex **80**. The same species was also synthesized starting from **78** and 2 equiv of Na­[OCAs] in the presence of 2.2.2-crypt. Theoretical investigations suggested that the mechanism for the formation of diarsallenide ligand in **80** is best described by a [2+2] cycloaddition reaction between coordinated [OCAs]^−^ and the coordinated cyarside ligand.^112^

**Figure 25:**
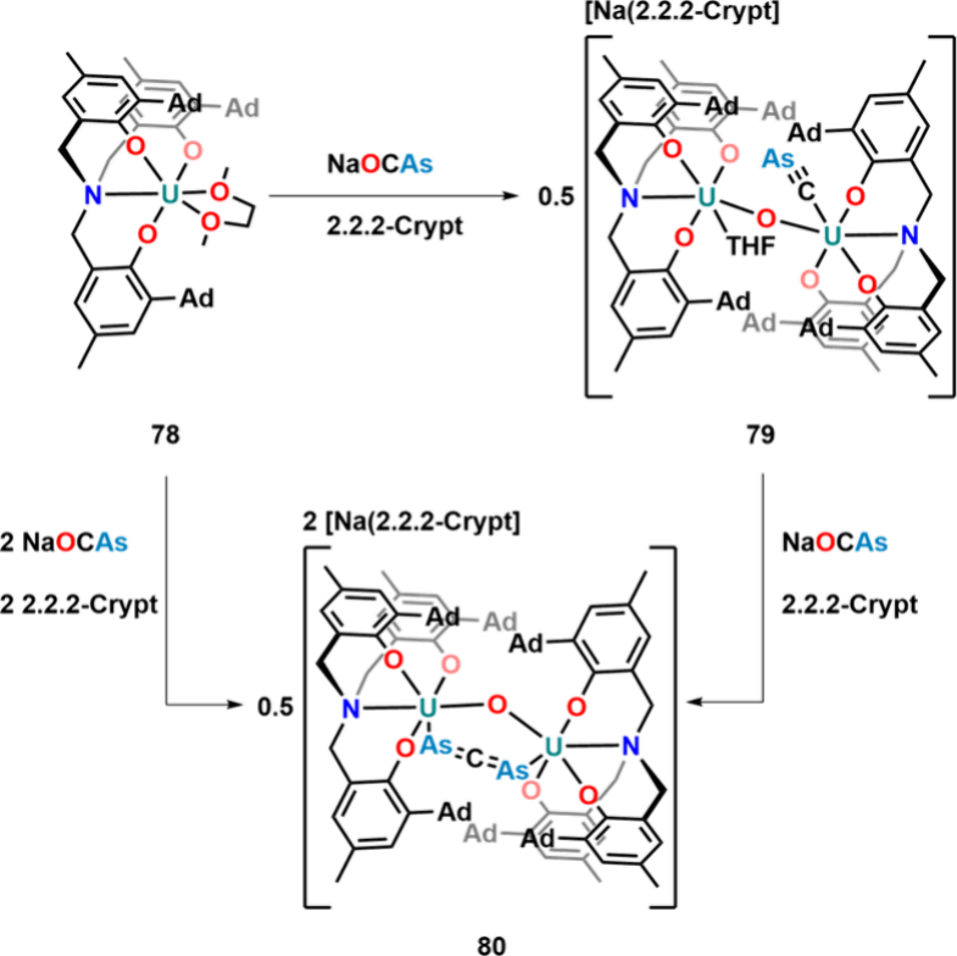
Synthesis of cyarside and bridging 1,3-diarsallenide ligands starting from Na­[OCAs] facilitated by uranium.

One year later, the κ^1^-O coordination of the [OCAs]^−^ was reported as complex **82**, starting from uranium­(IV) cation **81** (Figure 26).^114^ The stable coordination is insofar interesting as Meyer and co-workers also attempted the synthesis of a similar species, starting from [((Ad,MeArO)_3_N)­U­(DME)­(Cl)] and Na­[OCAs].^112^ However, in their case, only complicated mixtures were observed, highlighting the importance of the employed supporting ligand and synthetic strategy. Photolysis of complex **82** using a 125 W UV lamp resulted in decarbonylation of the [OCAs]^−^ ligand and formation of μ-η^2^,η^2^-As_2_H_2_-bridged complex **83**.^114^ The proton in this process is most likely delivered by solvent decomposition. The same complex was also synthesized starting from **81** and 1.4 equiv of KAsH_2_.^115^ Reduction of the complex with potassium graphite (KC_8_) in the presence of a sequestration reagent (2.2.2-crypt) resulted in the formation of complex **85** in which the [OCAs] moiety is trapped between two uranium centers. Computational investigations show that the uranium centers most likely adopt the +IV oxidation state with the [OCAs] moiety to be somewhere between di- and trianionic. A similar reaction was also reported with [OCP]^−^.^116^ The bent nature of the [OCAs]^−^ anion is further stabilized by extensive backbonding from the uranium atoms.^114^ In an attempt to obtain a neutral version of **85**, the authors also tried to reduce **82** with 1 equiv of uranium­(III) complex [U­(Tren^TIPS^)]. However, this resulted in complete decarbonylation, and μ-As diuranium complex **84** was observed instead, which was highly unstable in solution.

**Figure 26:**
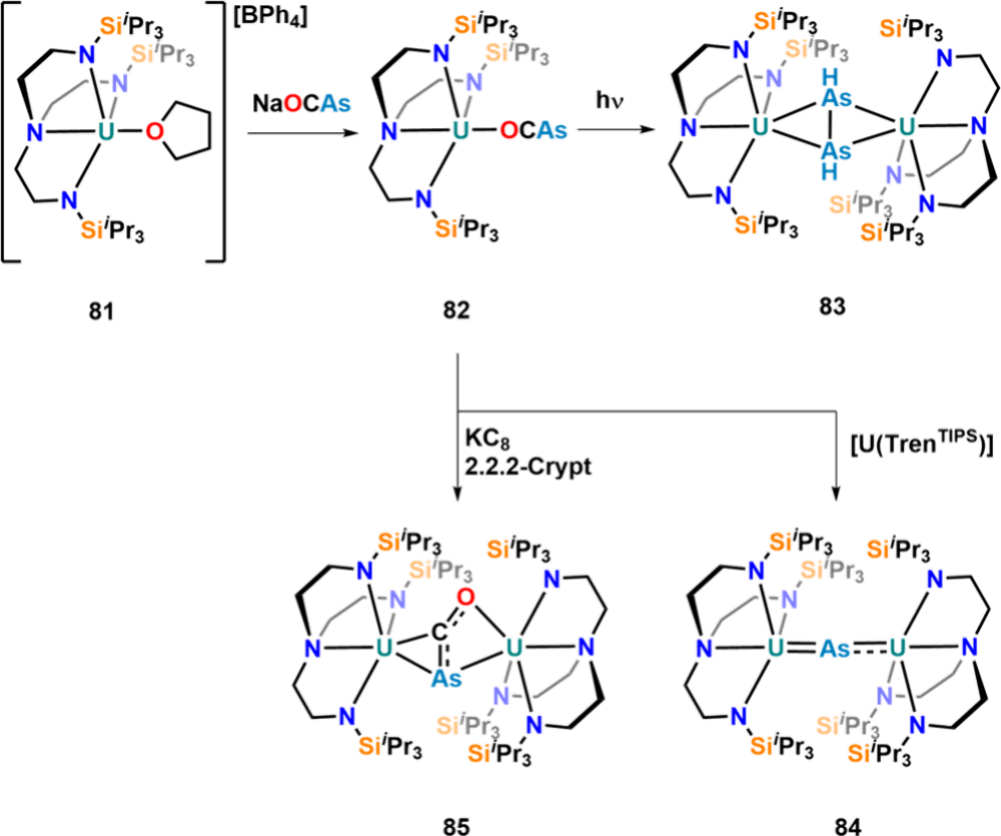
Reduction and photochemistry of κ^1^-O-coordinated [OCAs]^−^ anions on uranium­(IV).

#### [SCP]^−^, [SCAs]^−^, [SeCP]^−^, and [SeCAs]^−^: Coordination Chemistry

Switching to the heavier cyanates, namely, phospha- and arsaethynthiolate anions [SCP]^−^ and [SCAs]^−^, respectively, their chemistry has been barely explored to date. To the best of our knowledge, for phosphaethynthiolate anion [SCP]^−^, only three distinctive coordination compounds have been reported, while for the [SCAs]^−^ anion, only one report is present. Concurrently with its synthesis as stable potassium or sodium salts in 2015, the coordination of the [SCP]^−^ anion to low-valent tungsten(0) was also reported in the same publication (Figure 27).^48^ However, they found the phosphaethynthiolate anion to be highly ambiphilic,^300^ and a mixture of complexes **86-S** and **86-P** with κ^1^-sulfur (^31^P NMR δ −92.9 ppm) or κ^1^-phosphorus (^31^P NMR δ −192.6 ppm) coordination was observed. P coordination in **86-P** was concluded by the presence of ^173^W satellites (^1^ *J*_WP_ = 46 Hz) in the ^31^P NMR spectrum. Unfortunately, separation of the two coordination isomers was impossible, and both regioisomers were found to be unstable over a prolonged period of time in solution (1,2-dichlorobenzene; ca. 4 days). Theoretical calculations further proved the ambiphilic nature of the [SCP]^−^ anion. While common cyanates such as [OCN]^−^, [NCS]^−^, or even [OCP]^−^ display energies of 54.2, 31.4, or 47.5 kJ mol^–1^, respectively, for pnictide coordination over chalcogenide coordination, this value is only 3.5 kJ mol^–1^ for the [SCP]^−^ anion.^48^ However, one should keep in mind that for the ambiphilic cyanates, the ligated metal also plays an important role regarding the coordinating terminus.

**Figure 27:**
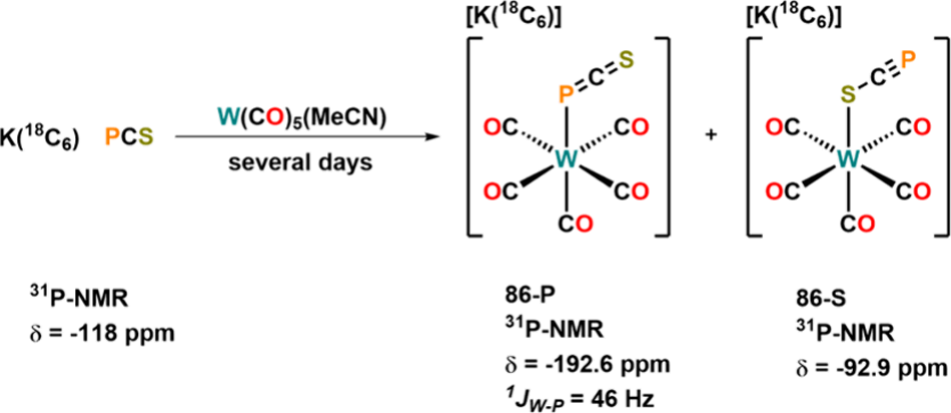
Unselective synthesis of two isomeric tungsten phosphaethynthiolate complexes showing either κ^1^-P or κ^1^-S coordination of the [SCP]^−^ anion.

Continuing to explore the chemistry of the [SCP]^−^ anion, Hohloch and Tambornino reported its unexpected η^3^ coordination (**88**) toward bis-anilidophosphine–lanthanum­(III) complex **87** (Figure 28).^117^ Theoretical calculations showed that the η^3^ coordination mode is preferred by 65 kJ mol^–1^ over the κ^1^-S coordination mode and by 75 kJ mol^–1^ over the κ^1^-P coordination mode. The complex showed a ^31^P NMR resonance at −44.9 ppm and was stable for weeks, and no signs of decomposition were observed in solution, as long as chlorinated solvents were avoided. If dichloromethane is introduced into the complex, slow conversion to starting chloride complex **87** was observed. The fate of the [SCP]^−^ fragment in this reaction remains unclear, but the reaction is a first indicator that the organic chemistry with the heavy cyanate anions might also be feasible. Further reactivity studies of complex **88** showed that it reacts selectively with cyclic alkyl amino carbenes (CAACs) undergoing a bond isomerization reaction remodeling the [SCP]^−^ anion to a κ^1^-S-bound [SPC=CAAC]^−^ anion in complex **89**, which can be formally understood as a sulfur/phosphorus analogue of a fulminate anion. The ^31^P NMR shift in this reaction changes from −44.9 ppm in **88** to 139.4 ppm in **89**, indicating a major change in the electronic situation around the phosphorus atom. Notably, this rearrangement reaction has also been observed for [OCP]^−^ using NHC ligands on both silicon^118^ and phosphorus.^119^ Further exploring the abundance of the η^3^ coordination mode, our group aimed for further metal fragments that undergo salt metathesis with Na­[SCP]. Similarly, we expanded our efforts toward the reactivity of the [SCAs]^−^ anion. We found that after the reaction between Na­[SCPn] (Pn = P or As) and tris-amide Zr­(IV) complex **90-I** a transient species was observed for both [SCP]^−^ and [SCAs]^−^ (**91-SC**Pn (Figure 29)).^120^ Albeit, so far this intermediate cannot be isolated. The similarity of the shift of −30.6 ppm in the ^31^P NMR spectrum of transient **91-SCP** (compared to −44.9 ppm in **88**) suggested that a similar η^3^ coordination was present. This is further supported by theoretical calculations showing the η^3^ coordination mode to be favored by ca. 20 kJ mol^–1^ over the κ^1^-S coordination mode for both anions in **91-SC**Pn (Pn = P or As). As mentioned, the η^3^-coordinated [SCP]^−^ and [SCAs]^−^ complexes of Zr­(IV) are not stable and selectively react to undergo the first [3+2] cycloaddition reaction involving these ions, forming new thio-thiadiphosphole or -diarsole ligands in the process. Notably, the regioselectivity of this reaction was controlled by solvent choice, and while reactions in THF gave access to 1,2-isomers **93-P** and **93-As**, reactions in toluene gave 1,3-isomer **92-P/92-As** as the sole product of the reaction for [SCP]^−^. In the case of the [SCAs]^−^ anion, the regioselectivity with respect to 1,3-isomer **92-As** was “diminished” and only a mixture of 1,2- and 1,3- isomers (53:47 **93-As**:**92-As**) was obtained. Theoretical calculations showed that the reaction follows a concerted [3+2] cycloaddition pathway, in which the transition state is slightly influenced by the solvent, explaining the observed regioselectivity. It should be noted at this point that the phosphaethynolate anion has shown a similar cycloaddition reactivity upon reaction with chloro-azolium salts^121^ or bromo-boranes.^122^ However, both reactions gave access to only one regioisomer (1,2-isomer), which in both cases is also not stable for prolonged times, even under inert conditions.

**Figure 28:**
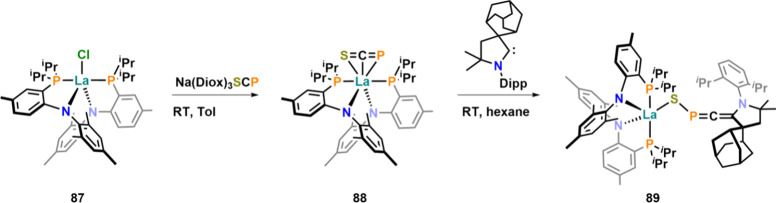
Unexpected η^3^-side-on coordination of the [SCP]^−^ anion at a lanthanum center and its CAAC-induced conversion into a heavy fulminate type anion.

**Figure 29:**
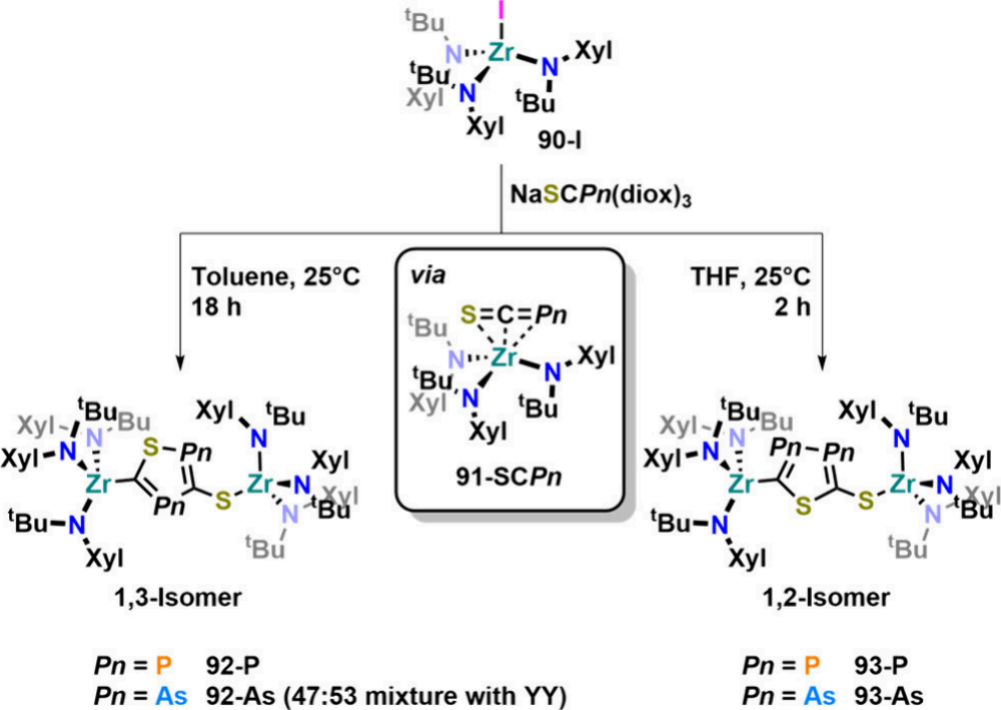
Formal [3+2] cycloaddition reactivity of the [SCP]^−^ and [SCAs]^−^ anions at a zirconium­(IV) amide center.

Notably, for the phosphorus and the arsenic analogues of the selenocyanate anion, i.e., phospha- and arsaethynselenolate anions [SeCP]^−^ and [SeCAs]^−^, respectively, no coordination chemistry has yet been reported.

## Conclusion and Future Perspectives

Despite being investigated for almost two centuries, this Review proves that cyanate anions are still of considerable interest in (inorganic) chemistry. In particular, the chemistry of heavy cyanates is currently blooming and still holds various exciting possibilities to be discovered. The difference in the electronic structure of these heavy cyanates compared to their “classical” congeners lays the foundation for the exploration of a completely new field of chemical conversions and the synthesis of a plethora of highly functionalized molecules and materials. These of course include the synthesis of yet unknown cyanate anions based on antimony or bismuth ([OCSb]^−^ or [OCBi]^−^, respectively), the synthesis of phosphorus and arsenic analogues of the tellurocyanate anion ([TeCP]^−^ or [TeCAs]^−^, respectively), and the further exploration of the main group and coordination chemistry of the known phosphorus and arsenic analogues of the thiocyanate and selenocyanate anions ([SCP]^−^, [SCAs]^−^, [SeCP]^−^, and [SeCAs]^−^). There are some future research questions. (I) Can these anions be used for the generation of new cyaphide or cyarside transfer reagents? (II) Can we extend the cycloaddition chemistry of these anions to facilitate heavy atom late-stage functionalization reactions? (III) Can these ions be used for radiolabeling reactions (using ^32^P nuclei)? (IV) Can these ions be used in organic chemistry?

The doors toward new and exciting cyanate chemistry are wide open. Given the vast success of the phosphaethynolate and the arsaethynolate anions (partly reviewed here, as well), we are certain that the heavier analogues will join them and become valuable synthons and building blocks across the whole periodic table.

## Footnotes

a The synthesis of Shober and Spanutius from 1894 was likely the first synthesis of [OCP]^−^; however, the limitations of the time did not allow for the analysis and assignment of the correct chemical formula.

b “Blaustoff-Wasserstoffsäure” in German or “bleu de Prusse” in French.

c Porrett writes, “In reflecting on the means most likely to attain this end, it occurred to me, that I should succeed if I decomposed prussiate of iron by double electrive attraction rather than by single, employing, instead of a pure potash, that alkali, in combination with a substance uniting the properties of solubility when combined with potash, strong attraction for oxide of iron and insolubility when united to that oxide. The only substances I could think of possessing all of these requisite properties were the succinic acid and sulfur; as the high price of the former precluded its use for this purpose, I determined to employ the latter.”

d Also, within his work he explains that the new principle comprises the same elements as prussic acid plus sulfur, and thus he calls the thiocyanates “sulphuretted chyazates”.

e “Blaustoff” in German or “cyanogène” in French.

f “Lässt man Blaustoff and Wasser über das zweite Bleioxyd (Mennige) oder über das höchste Manganoxyd (schwarzem Braunstein) stehen, so wird er allmählig ganz and gar eingesogen, and das Wasser verliert selbst allen Geruch; nach dem Filtrierern ist es citronengelb and merkbar neutral. [···] Da sich aber beim Einwirken von Blaustoff auf nasse Mennige Ammoniak bildet, so muss dabei Wasser zersetzt werden and es ist die Frage, was aus dem Sauerstoff dieses Wassers wird, and welches die Säure ist, die die alkalische Auflösung neutraliziert? Sollte sich hierbei etwa Blaustoffsäure gebildet haben?”

g Crookes elemental analysis is typical for its time. For diatomic gases, the atomic weights are often assumed to be twice the weight. This prevailed until a unified system was introduced.

h In Becker’s original synthesis, [(TMS)_2_P]^−^ and dimethyl carbonate were used.

i The literature value of 1.07(1) Å for the N–C bond length in [PPN]­[TeCN] is likely too short due to the processing of data without absorption correction.

j
^13^C referenced to (CH_3_)_4_Si, ^14^N and ^15^N to MeNO_2_, ^31^P to 85% H_3_PO_4_, ^77^Se to Me_2_Se, and ^125^Te to Me_2_Te (90%) in C_6_D_6_. Chemical shifts (parts per million) and references. [OCN]^−^: ^13^C 129.7,^57^ ^14^N −303.8.^58^ [SCN]^−^: ^13^C 134,^57,59^ ^14^N −173.8.^60–62^ [SeCN]^−^: ^13^C 120.8,^59,63^ ^14^N −139.8,^62^ ^77^Se −314.^63^ [TeCN]^−^: ^13^C 77.8,^39^ ^15^N −94.3,^39^ ^125^Te −566.^39^ [OCP]^−^: ^13^C 166.6,^42^ ^31^P −384.2.^42^ [SCP]^−^: ^13^C 193.6,^47,51^ ^31^P −120.9.^47,51^ [SeCP]^−^: ^13^C 189.3,^51^ ^31^P −46.9,^51^ ^77^Se 84.3.^51^ [OCAs]^−^: ^13^C 179.4.^50^ [SCAs]^−^: ^13^C 210.0.^51^ [SeCAs]^−^: ^13^C 206.0,^51^ ^77^Se 248.8.^51^
